# Adenylosuccinate Synthase 1 Deficiency Improves Energy Metabolism by Promoting Adipose Tissue Re‐esterification via Glycerol Kinase Upregulation

**DOI:** 10.1002/advs.202506270

**Published:** 2025-10-21

**Authors:** Jingjing Sun, Miriayi Alimujiang, Wenfei Li, Shuqin Chen, Yingying Su, Tingting Hu, Xuhong Lu, Yafen Ye, Ningning Bai, Fan Hu, Xiaoya Li, Rongrong Xu, Jun Xu, Jiarui Zhao, Yan Lu, Xiaojing Ma, Ying Yang

**Affiliations:** ^1^ Department of Endocrinology and Metabolism Shanghai Diabetes Institute Shanghai Clinical Center for Diabetes Shanghai Key Laboratory of Diabetes Mellitus Shanghai Key Clinical Center for Metabolic Disease Shanghai Sixth People's Hospital Affiliated to Shanghai Jiao Tong University School of Medicine Shanghai 200233 China; ^2^ Department of Endocrinology and Metabolism Shanghai Geriatric Medical Center Shanghai 201100 China; ^3^ Department of Endocrinology and Metabolism Zhongshan Hospital Fudan University Shanghai 200032 China; ^4^ Department of Endocrinology and Metabolism The First Affiliated Hospital of Ningbo University Ningbo Zhejiang 315010 China; ^5^ Department of Endocrinology Shenzhen Second People's Hospital the First Affiliated Hospital of Shenzhen University Health Science Center of Shenzhen University Shenzhen Clinical Research Center for Metabolic Diseases Shenzhen Center for Diabetes Control and Prevention Shenzhen Guangdong 518035 China; ^6^ Department of Traditional Chinese Medicine Shanghai Sixth People's Hospital Affiliated to Shanghai Jiao Tong University School of Medicine Shanghai 200233 China; ^7^ College of Biological Science and Medical Engineering Donghua University Shanghai 200051 China; ^8^ Institute of Metabolism and Regenerative Medicine Digestive Endoscopic Center Shanghai Sixth People's Hospital Affiliated to Shanghai Jiao Tong University School of Medicine Shanghai 200233 China

**Keywords:** adipose thermogenesis, Adss1, glycerol kinase, HDAC activity, obesity, re‐esterification

## Abstract

Purine metabolism enzymes have a well‐established role in maintaining the nucleotide pool, thereby sustaining cellular energy homeostasis. Although reduced purine nucleotide concentrations have been reported can influence uncoupling protein 1 (UCP1)activity in thermogenic adipocytes, our study identifies adenylosuccinate synthase 1 (Adss1), an enzyme in de novo purine biosynthesis, as a critical regulator of metabolic remodeling in inguinal white adipose tissue (iWAT) through a mechanism distinct from UCP1 activity. Adipose‐specific Adss1 knockout mice showed increased energy expenditure and resistance to diet‐induced obesity with improved metabolic dysfunction. Loss of Adss1 upregulates glycerol kinase (Gk) expression, thereby stimulating glycerol‐dependent fatty acid re‐esterification in iWAT. This adaptation prevents lipotoxic accumulation of free fatty acids and drives lipid synthesis–oxidation cycling, activating thermogenic programs. The Adss1 deficiency‐driven iWAT browning and re‐esterification are abolished in adipose‐specific Adss1 and Gk double‐knockout mice, confirming the functional dependence on Gk. Mechanistically, Adss1 interacts with histone deacetylase 3 (HDAC3) in the cytosol of beige adipocytes, altering its nucleo‐cytoplasmic distribution. Adss1 deficiency reduced nuclear HDAC3 and increased cytosolic pools, which suppresses HDAC activity and enhances histone H3 lysine 27 acetylation at the Gk promoter, elevating Gk expression. Collectively, our findings reveal an unrecognized role of Adss1 in adipose physiology, highlighting its potential as a regulator of adipose energy metabolism.

## Graphical abstract

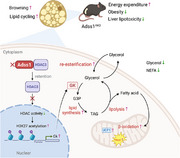

## 1 Introduction

Obesity results from an imbalance in energy homeostasis, characterized by energy intake exceeding energy expenditure.^[^ ^1^, ^2^ ^]^ White adipose tissue (WAT) plays a pivotal role in maintaining systemic energy balance by regulating lipid storage and mobilization,^[^ ^3^ ^]^ as well as mediating the transformation of white adipocytes into beige adipocytes.^[^ ^4^ ^]^ Under conditions of energy surplus, excess nutrients are stored as triglycerides (TG) in WAT.^[^ ^3^ ^]^ However, excessive lipid accumulation can lead to lipotoxicity, causing adipose tissue dysfunction and subsequently contributing to metabolic disorders such as insulin resistance,^[^ ^5^, ^6^ ^]^ non‐alcoholic fatty liver disease,^[^ ^7^ ^]^ and metabolic cardiomyopathy.^[^ ^8^ ^]^ Conversely, during periods of energy demand, such as fasting or exercise, TG in the WAT are broken down to produce non‐esterified fatty acids (NEFA) and glycerol. These NEFA may be directly oxidized within adipocytes or exported as fuel for other tissues, such as the liver and muscles.^[^ ^9^ ^]^ Nonetheless, a significant proportion of NEFA is reabsorbed by adipocytes and re‐esterified into TG, which, along with lipolysis, is referred to as the re‐esterification cycle (also termed the futile lipid cycle). This cycle not only dissipates energy as heat through ATP consumption but also maintains the dynamic balance of fatty acids inside and outside the cell, which is crucial for ensuring proper adipocyte function and detoxifying excess free fatty acids.^[^ ^10^, ^11^ ^]^ Research has shown that ≈40% of released fatty acids in human white adipocytes are recycled and stored in lipid droplets.^[^ ^12^ ^]^ Moreover, compared to individuals of normal weight, obese individuals exhibit significantly reduced lipid cycling.^[^ ^13^ ^]^ Notably, under cold exposure and PPARγ agonists, the re‐esterification cycle is activated,^[^ ^11^, ^14^ ^]^ which also promotes the browning of white adipose tissue.^[^ ^15^ ^]^ However, the relationship between the re‐esterification cycle and the browning of white adipose tissue remains unclear, and its potential role in the development of obesity has yet to be fully elucidated.

Purine nucleotide metabolism includes biosynthesis and degradation, with biosynthesis proceeding through two pathways: the de novo synthesis pathway and the salvage pathway.^[^ ^16^ ^]^ Under the regulation of purine metabolic enzymes, the levels of purine nucleotides are maintained to modulate cellular functions. In the context of energy metabolism, cellular inhibitory nucleotides (such as ADP, ATP, GTP, and GDP) can suppress uncoupling protein 1 (UCP1) activity.^[^ ^17^, ^18^, ^19^ ^]^ Salvatore et al. first observed that the guanosine monophosphate reductase (GMPR), an enzyme involved in purine nucleotide degradation, was upregulated in the brown adipose tissue (BAT) of rats following cold exposure.^[^ ^20^ ^]^ Likewise, Li et al. found that cold exposure in mice induces the upregulation of GMPR and AMP deaminase in BAT. Furthermore, they demonstrated that overexpression of GMPR led to a reduction in suppressive purine nucleotide levels and an increase in UCP1 activity in the UCP1‐expressing human embryonic kidney cells.^[^ ^21^ ^]^ Although many enzymes are known to be involved in the biosynthesis, interconversion, and degradation of purine compounds, their exact functions in adipocytes remain unclear—particularly whether they can modulate thermogenesis through mechanisms beyond UCP1 activity. Additionally, their potential roles in systemic energy homeostasis and their impact on metabolic disorders, such as obesity, require further investigation.

Adenylosuccinate synthase is a key enzyme in de novo purine nucleotide biosynthesis and catalyzes the conversion of inosine monophosphate (IMP) to adenylosuccinic acid (S‐AMP). This enzyme exists in two isoforms across all studied mammalian species: the basic isoform (Adss1), which is predominantly found in muscle tissues, and the acidic isoform (Adss2), which is widely distributed among various tissues.^[^ ^22^, ^23^ ^]^ To date, most basic research has primarily focused on Adss as a purine nucleotide synthase, whereas the role of Adss1 remains relatively underexplored, with only a few clinical studies have mentioned that mutations in Adss1 gene can lead to myopathy, a congenital metabolic disorder inherited in an autosomal recessive manner, characterized by hypotonia, progressive muscle weakness, and lipid accumulation in muscle tissue. Additionally, recent studies have also found that Adss1 is involved in regulating cardiomyocyte proliferation and cardiac regeneration.^[^ ^24^, ^25^, ^26^, ^27^ ^]^ Currently, no studies have reported on the role of Adss1 in adipose tissue, and its potential contribution to adipose biology remains largely unexplored.

In this study, we found that Adss1 was significantly induced in iWAT in response to diverse adaptive thermogenic stimuli. Additionally, a significant correlation was identified between the expression of Adss1 (the human homolog, ADSS1) and genes related to thermogenesis and mitochondrial oxidative phosphorylation in human adipose tissue, as well as with key clinical metabolic parameters. The specific deletion of Adss1 in adipose tissue reshaped the function of iWAT, promoting its browning and enhancing energy expenditure in mice. Moreover, the loss of Adss1 increased re‐esterification in iWAT, leading to a reduction in serum NEFA and glycerol levels, which alleviated lipotoxicity under conditions of nutritional overload. Furthermore, we identified glycerol kinase (Gk) as a key downstream effector of Adss1. Mechanistically, cytosolic Adss1 binds histone deacetylase 3 (HDAC3) and alters its nuclear–cytoplasmic distribution in beige adipocytes; accordingly, Adss1 deficiency reduces nuclear HDAC3 and increases cytosolic HDAC3, a shift associated with altered HDAC activity. Consistent with this mechanism, Adss1 knockout increased histone H3 lysine 27 (H3K27) acetylation at the Gk promoter, which correlates with upregulated Gk expression and promotes re‐esterification cycles in iWAT. Importantly, the induction of iWAT browning and re‐esterification in Adss1^AKO^ mice was reversed in adipose tissue‐specific Adss1 and Gk double knockout mice. Collectively, our data reveal a previously unrecognized role for Adss1 in regulating adipose tissue lipid processing and energy balance, distinct from its classical role in de novo purine nucleotide biosynthesis.

## 2 Results

### 2.1 Induction of Adipose Adss1 Expression in Response to Adaptive Thermogenesis

**Figure 1:**
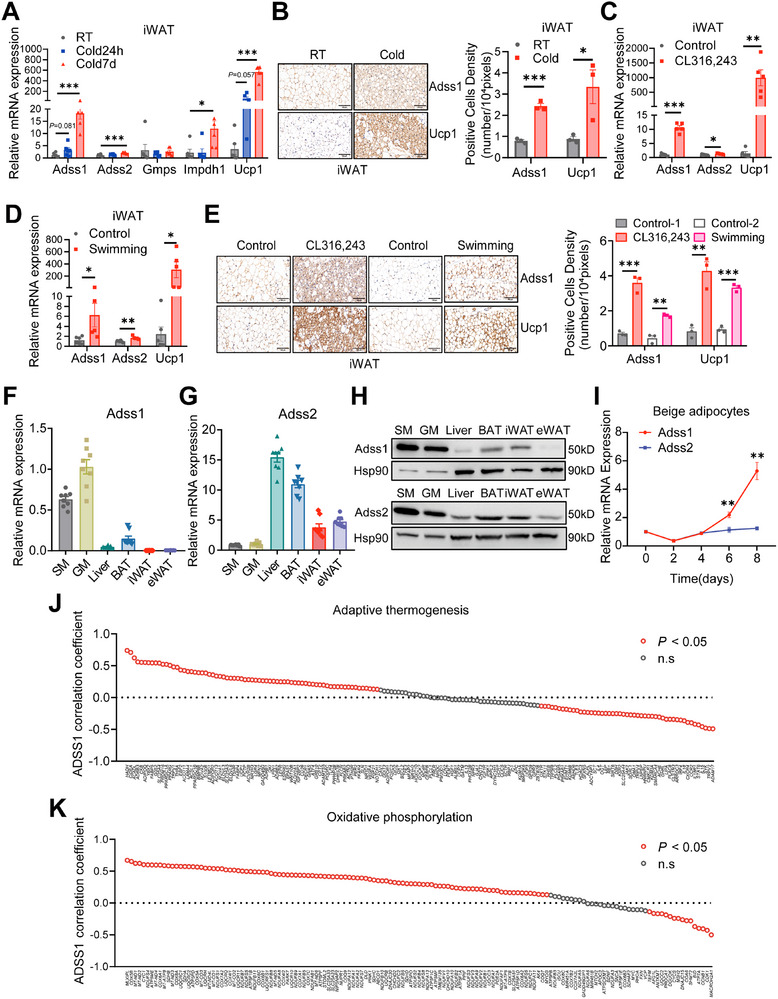
Induction of Adss1 expression in adipose tissue in response to adaptive thermogenesis. A) mRNA expression of Adss1, Adss2, Gmps, Impdh1, and Ucp1 in iWAT from WT mice housed at RT or exposed to cold for 24 h or 7 days (*n* = 6). B) Representative images of immunohistochemical staining of Adss1 and Ucp1 in iWAT from WT mice housed at RT or after 7 days of cold exposure, scale bar = 100 µm. C–E) Relative mRNA expression of the indicated genes (C,D), and representative images of immunohistochemical staining (E) in iWAT from WT mice treated with CL316243 for 7 days (*n* = 5) or subjected to swimming exercise for 14 days (control *n* = 6, swimming *n* = 5), scale bar = 100 µm. F–H) Expression of Adss1 (F), Adss2 (G) at the mRNA level, and protein expression (H) in various tissues from WT mice housed at RT (*n* = 8). SM, soleus muscle; GM, gastrocnemius. I) Time‐course of Adss1 and Adss2 mRNA expression during beige adipocyte differentiation (*n* = 3), shown relative to day 0. J,K) Correlation between ADSS1 expression and genes related to adaptive thermogenesis (J), or oxidative phosphorylation (K) in VAT. Significantly correlated transcripts (*P* < 0.05) are indicated in red. Data are presented as mean ± SEM. Statistical significance was assessed using Student's t‐test (A–E, and I) and Pearson's two‐tailed correlation analysis (J, K). ^*^ *p* < 0.05, ^**^ *p* < 0.01, ^***^ *p* < 0.001.

To investigate the transcriptional regulation of purine nucleotide metabolism enzymes in cold‐activated adipose tissues, wild‐type (WT) C57BL/6J male mice were exposed to 4 °C for 24 h and 7 days. Our analysis revealed distinct expression patterns in BAT and iWAT. Consistent with previous studies,^[^ ^20^, ^21^ ^]^ the expression of the catabolic enzymes *Gmpr* and *Gda* was significantly upregulated in both BAT (Figure S1A, Supporting Information) and iWAT (Figure S1B, Supporting Information) following cold exposure. It's noteworthy that cold exposure induced substantial alterations in the expression of purine nucleotide biosynthetic enzymes. Specifically, both Adss1 and Adss2 showed increased expression in iWAT (**Figure** **1**A) and BAT (Figure S1C, Supporting Information). Notably, iWAT exhibited a particularly robust response, with Adss1 expression increasing nearly 20‐fold after 7 days of cold exposure compared to control mice, whereas Adss2 displayed only moderate upregulation (Figure 1A). These transcriptional changes were further validated at the protein level, as immunohistochemical analysis confirmed elevated Adss1 protein levels in iWAT (Figure 1B). In contrast, BAT showed a more modest response, with only slight increases in Adss1 and Adss2 expression after cold exposure (Figure S1C,D, Supporting Information). These findings highlight the tissue‐specific transcriptional regulation of purine metabolic enzymes during cold adaptation, with iWAT demonstrating a particularly pronounced upregulation of Adss1.

We further examined Adss1 expression patterns under two distinct physiological conditions: β‐adrenergic stimulation and exercise training. Following a 7‐day treatment with the β‐adrenergic agonist CL316243, we observed a striking tenfold upregulation of Adss1 expression in iWAT, whereas Adss2 exhibited only a marginal 1.3‐fold increase (Figure 1C). In contrast, in BAT, Adss1 expression remained unchanged, while Adss2 showed a moderate 1.5‐fold upregulation (Figure S1E, Supporting Information). Similarly, after 14 days of swimming training, Adss1 mRNA levels were significantly elevated in iWAT, whereas Adss2 exhibited only a slight increase (Figure 1D). In BAT, both isoenzymes displayed modest upregulation following exercise training (Figure S1F, Supporting Information). These transcriptional changes were further corroborated at the protein level, as Adss1 protein expression was markedly upregulated in iWAT under both CL316243 stimulation and swimming training conditions (Figure 1E). Examining tissue distribution patterns, we found that Adss1 expression was predominantly localized to muscle tissue, whereas Adss2 exhibited a more ubiquitous expression profile across various tissues (Figure 1F–H). Notably, during adipocyte differentiation, Adss1 expression showed a significant increase in mature beige adipocytes compared to Adss2 (Figure 1I).

To explore the clinical relevance of our findings, we analyzed RNA‐seq data from adipose tissues of 236 lean and obese individuals.^[^ ^28^ ^]^ Given that human visceral adipose tissue (VAT) is characterized by higher expression of beige adipocyte marker genes and enhanced mitochondrial oxidative phosphorylation compared to subcutaneous fat, we focused our analysis on VAT. ADSS1 expression in VAT was significantly associated with genes involved in adaptive thermogenesis (Figure 1J) and mitochondrial oxidative phosphorylation (Figure 1K). Collectively, these findings strongly suggest that Adss1 is intricately linked to thermogenic processes in adipose tissue and may play a pivotal role in regulating energy metabolism.

### 2.2 Adss1^AKO^ Mice Stimulate the Browning of White Adipose Tissue and Energy Expenditure

**Figure 2:**
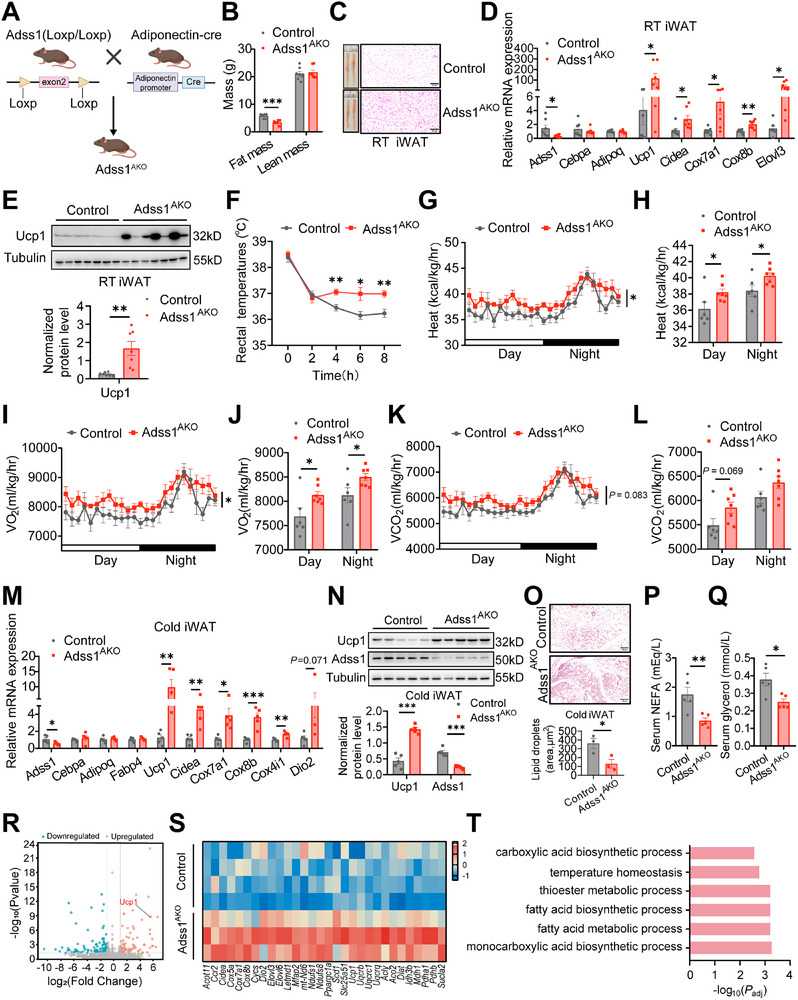
Browning of white adipose tissue and increased energy expenditure in Adss1^AKO^ mice. A) Schematic diagram of the Adss1^AKO^ mouse model. B–E) Analysis of metabolic parameters in 30‐week‐old control and Adss1^AKO^ mice fed CD and housed at RT (*n* = 7), including body composition (B), overall and representative H&E staining of iWAT, scale bar = 100 µm (C), mRNA expression of Adss1, adipocyte marker genes and thermogenic genes (D), and Ucp1 protein levels in iWAT (E). F) Rectal core body temperature of 8‐week‐old control and Adss1^AKO^ mice exposed to cold (5 °C) at indicated time points (*n* = 6). G–L) Indirect calorimetry of 9‐week‐old control (*n* = 6) and Adss1^AKO^ (*n* = 7) mice following 3 days of cold exposure, including heat production curve (G), average heat production (H), VO_2_ curve (I), average VO_2_ (J), VCO_2_ curve (K) and average VCO_2_ (L) during both light and dark cycles. M–Q) Metabolic analysis of control and Adss1^AKO^ mice after 3 days of cold exposure (*n* = 5), including mRNA expression of adipocyte marker and thermogenic genes (M), representative Western blot and quantification of Ucp1 and Adss1 protein levels in iWAT (N), H&E staining and quantification of lipid droplets area of iWAT, scale bar = 100 µm (O), serum NEFA (P) and glycerol (Q). R–T) Transcriptomic profiling of iWAT from control (*n* = 4) and Adss1^AKO^ (*n* = 3) mice housed at RT on a chow diet. Scatter plot displaying differentially expressed genes in iWAT (Fold change > 2, FDR < 0.05) (R). Heatmaps showing upregulated genes (Adss1^AKO^ mice versus control) involved in thermogenesis, oxidative phosphorylation, and the TCA cycle (*P* < 0.05) (S). Enriched biological pathways upregulated in iWAT of Adss1^AKO^ mice (Fold change > 1.5, FDR < 0.05) (T). Data are presented as mean ± SEM. ^*^ *p* < 0.05, ^**^ *p* < 0.01, ^***^ *p* < 0.001. Student's *t*‐test (B, D‐F, H, J, L‐N, O, P, and Q). Two‐way ANOVA (G, I, and K).

To investigate the physiological role of Adss1 in adipose tissue function and systemic metabolism, we generated adipose‐specific Adss1 knockout (Adss1^AKO^) mice using adiponectin‐Cre mice (**Figure** **2**A; Figure S2A,B, Supporting Information). Selective deletion of Adss1 in the BAT, iWAT, and epididymal white adipose tissue (eWAT) was confirmed using qPCR and Western blotting in Adss1^AKO^ mice compared with littermate controls (Figure S2C–F, Supporting Information). Under chow diet (CD) feeding at room temperature (RT), Adss1^AKO^ mice exhibited reduced fat mass (Figure 2B) and decreased fat depot weight (Figure S3A, Supporting Information), despite no significant difference in body weight (Figure S3B, Supporting Information). Food intake was comparable between the groups (Figure S3C, Supporting Information). Notably, iWAT from Adss1^AKO^ mice displayed a reddish appearance, and histological analysis revealed smaller, multilocular adipocytes (Figure 2C). Molecular characterization showed that Adss1^AKO^ mice activated a brown/beige fat thermogenic gene program in iWAT, as evidenced by the upregulation of thermogenic markers (*Ucp1*, *Cidea*, *Cox7a1*, and *Cox8b*), while the expression of adipocyte differentiation markers *Cebpa* and *Adipoq* remained unchanged (Figure 2D). Consistently, Ucp1 protein levels were significantly increased in Adss1^AKO^ mice (Figure 2E). Importantly, Adss1^AKO^ mice showed improved glucose tolerance (Figure S3D,E, Supporting Information), while insulin sensitivity exhibited a non‐significant but suggestive trend toward improvement (*P* = 0.09) (Figure S3F,G, Supporting Information), with the expression of glucose uptake genes (*Slc2a1* and *Slc2a4*) in Adssl1 knockdown beige adipocytes remaining unchange (Figure S3H, Supporting Information). These findings suggest that adipose‐specific deletion of Adss1 promotes a thermogenic phenotype and enhances glucose metabolism without affecting overall body weight or insulin sensitivity.

Next, we assessed the cold tolerance of Adss1^AKO^ mice by monitoring the rectal temperature at 5 °C in a cold chamber. Adss1^AKO^ mice maintained core body temperature more effectively than control mice during cold exposure (Figure 2F). Consistently, heat production, oxygen consumption (VO_2_), and carbon dioxide production (VCO_2_) in Adss1^AKO^ mice were higher upon cold exposure for 3 days compared with the respective parameters in control mice (Figure 2G–L; Figure S3I–K, Supporting Information), as detected using indirect calorimetry. No apparent alterations in the respiratory exchange ratio (RER) (Figure S3L, Supporting Information) or physical activity (Figure S3M–O, Supporting Information) were detected between the two groups. Under cold exposure, the expression of thermogenic genes, including *Ucp1, Cox8b*, and *Cidea*, was increased in the iWAT of Adss1^AKO^ mice (Figure 2M), along with increased protein levels of Ucp1 (Figure 2N). In addition, no differences were observed in the levels of general adipogenic markers *Cebpa*, *Adipoq*, and *Fabp4* (Figure 2M). H&E staining revealed that the Adss1^AKO^ mice displayed a higher density of multilocular adipocytes in the iWAT after cold exposure for 3 days compared with the littermate controls (Figure 2O). Moreover, after 7 days of cold exposure, the Adss1^AKO^ mice maintained elevated expression levels of thermogenic genes, such as *Ucp1* and *Cox8b* (Figure S3P, Supporting Information). In addition, serum NEFA and glycerol levels decreased in the Adss1^AKO^ mice upon cold exposure (Figure 2P,Q).

To characterize the transcriptome, RNA sequencing was conducted on iWAT from control and Adss1^AKO^ mice maintained on a CD at RT. A total of 74 genes were significantly upregulated, while 101 genes were markedly downregulated in Adss1^AKO^ mice compared with those in control mice (Figure 2R). Notably, most genes related to thermogenesis, oxidative phosphorylation, and the tricarboxylic acid (TCA) cycle were upregulated (Figure 2S). Gene ontology analysis further confirmed that the most enriched pathways in Adss1^AKO^ iWAT were related to thermogenesis and fatty acid metabolism (Figure 2T).

Given the increased energy expenditure observed in Adss1^AKO^ mice, which could also be attributed to enhanced BAT activity, we examined the thermogenic and lipid metabolic profile of BAT in these mice. Notably, we observed a noticeable upregulation of Ucp1 protein levels in the BAT of Adss1^AKO^ mice only upon acute cold challenge for 6 h (Figure S4A, Supporting Information). However, under RT conditions (Figure S4B–D, Supporting Information) and prolonged cold exposure (Figure S4E–G, Supporting Information), the expression of brown fat markers (*Ucp1* and *Cidea*) and genes involved in lipid metabolism remained unchanged. These results indicate that enhanced energy expenditure in Adss1^AKO^ mice is primarily driven by the promotion of iWAT browning.

### 2.3 Adss1 Regulates the Thermogenic Program in Beige and Brown Adipocytes

**Figure 3:**
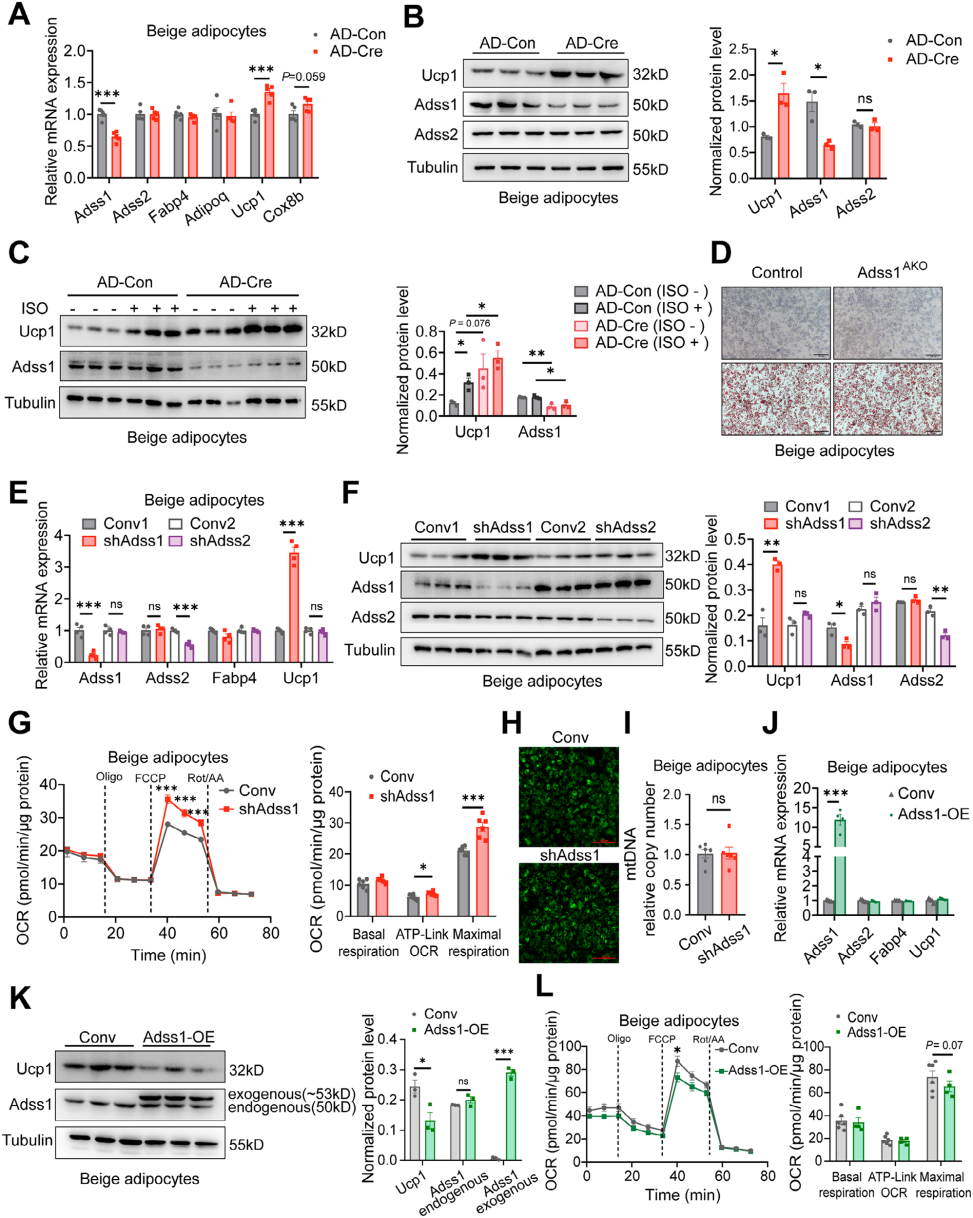
In vitro studies of Adss1 function in beige adipocytes. A) mRNA expression of the indicated genes in differentiated beige adipocytes following Adss1 deletion via infection with Cre recombinase adenovirus (AD‐Cre) (*n* = 5). B) Immunoblot detection and quantification of Ucp1, Adss1, and Adss2 protein levels after Adss1 knockdown in beige adipocytes (*n* = 3). C) Western blot and quantification of Ucp1 expression under basal conditions and following 12 h stimulation with 5 µM ISO in Adss1‐deficient beige adipocytes (*n* = 3). D) Microscopic imaging of cell morphology and Oil Red O staining in differentiated beige adipocytes derived from control and Adss1^AKO^ mice, scale bar = 100 µm. E,F) mRNA expression (E, *n* = 4) and protein levels (F, *n* = 3) of Ucp1 in beige adipocytes infected with shAdss1 or shAdss2 lentivirus. G) Mitochondrial respiration was assessed and quantification in Adss1 knockdown and scramble beige adipocytes under various drug treatments (*n* = 6). H–I) Mitochondrial content assessed using mitotracker staining, scale bar = 50 µm (H) and mtDNA quantification (I*, n* = 6) following Adss1 knockdown. J–L) mRNA expression (J, *n* = 4), protein levels (K, *n* = 3), and mitochondrial respiration (L, *n* = 5) in control and Adss1 overexpressing beige adipocytes. Data are presented as mean ± SEM. ^*^ *P* < 0.05, ^**^ *p* < 0.01, ^***^ *p* < 0.001. Student's *t*‐test (A‐C, E‐G, I‐L).

To investigate the cell‐autonomous role of Adss1 in the regulation of the thermogenic program, we performed gain‐ and loss‐of‐function studies in differentiated mature beige and brown adipocytes. Stromal vascular fractions (SVFs) of iWAT and BAT were isolated from Adss1^fl/fl^ mice and differentiated into beige or brown adipocytes. Then, the differentiated adipocytes were infected with Cre recombinase adenovirus to silence Adss1 expression. Adss1 knockdown significantly upregulated the expression of the thermogenic marker Ucp1 at both the gene and protein levels (**Figure** **3**A,B), with a further increase upon isoproterenol (ISO) stimulation (Figure 3C) in beige adipocytes. These effects were not due to enhanced adipogenesis, as differentiated beige adipocytes isolated from control and Adss1^AKO^ mice exhibited similar differentiation capacity (Figure 3D) and comparable expression of adipogenic genes (Figure S5A, Supporting Information) in vitro. Moreover, differentiated beige adipocytes isolated from Adss1^AKO^ mice also exhibited enhanced thermogenic function (Figure S5A,B, Supporting Information). Consistently, similar changes in thermogenic genes and proteins were observed in brown adipocytes following Adss1 knockdown (Figure S5C,D, Supporting Information). In contrast, silencing Adss2 had no effect on the thermogenic program (Figure 3E,F).

To assess mitochondrial function, we performed extracellular flux analysis using a Seahorse analyzer. Adss1 knockdown significantly increased maximal mitochondrial respiratory capacity in beige adipocytes (Figure 3G). However, there were no differences in mitochondrial number or mitochondrial DNA (mtDNA) content (Figure 3H,I), indicating that Adss1 regulates mitochondrial respiration independently of mitochondrial abundance. In contrast to the knockdown results, overexpression of Adss1 in beige adipocytes led to Ucp1 suppression without affecting adipocyte differentiation marker genes expression (Figure 3J,K), and also inhibited mitochondrial respiration (Figure 3L). Similar results were obtained for the thermogenic program in brown adipocytes following Adss1 overexpression (Figure S5E, Supporting Information).

### 2.4 Adss1^AKO^ Mice Protect Against Diet‐Induced Obesity and Metabolic Disorders

**Figure 4:**
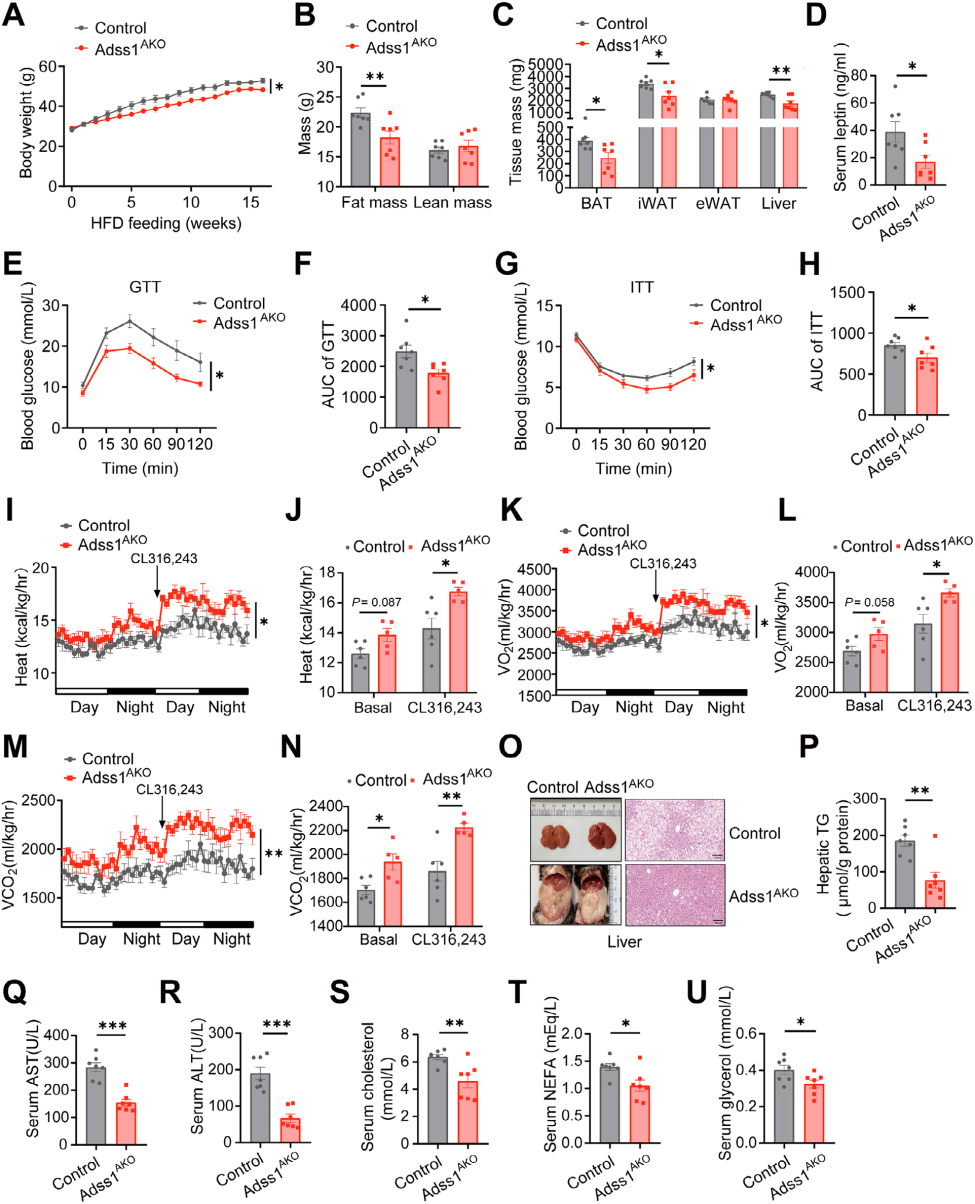
Metabolic phenotyping of Adss1^AKO^ mice under HFD‐feeding. Metabolic analysis of age‐matched littermate control and Adss1^AKO^ mice after 16 weeks of HFD feeding. A) Body weight (*n* = 7). B) Body composition (*n* = 7). C) Tissue weight (*n* = 7). D) Serum leptin (*n* = 7). E,F) GTT and corresponding area under the curve (AUC) analysis (*n* = 7). G,H) ITT and AUC analysis (*n* = 7). I–N) Metabolic cage analysis of HFD‐fed control (*n* = 6) and Adss1^AKO^ (*n* = 5) mice under basal and 1 mg kg^−1^ CL316243 treatments, including heat production curve (I) and average heat production (J), VO_2_ curve (K) and average VO_2_ (L), VCO_2_ curve (M) and average VCO_2_ (N). O–P) Representative H&E staining of liver tissue, scale bar = 100 µm (O) and hepatic TG (P) after HFD. Q–U) Serum AST (Q) and ALT (R), total cholesterol (S), NEFA (T), and glycerol (U) levels (*n* = 7). Data are presented as mean ± SEM. ^*^ *p* < 0.05, ^**^ *p* < 0.01, ^***^ *P* < 0.001. Two‐way ANOVA (A, E, G, I, K, and M). Student's *t*‐test (B‐D, F, H, J, L, N, and P‐U).

Having established that conditional ablation of Adss1 enhances whole‐body energy expenditure in CD‐fed mice, we next examined whether Adss1^AKO^ mice were protected from diet‐induced obesity. When challenged with a high‐fat diet (HFD) for 16 weeks, Adss1^AKO^ mice gained less weight compared to their littermate controls (**Figure** **4**A), despite comparable food intake between the two groups (Figure S6A, Supporting Information). Magnetic resonance imaging confirmed that Adss1^AKO^ mice had reduced fat mass compared to controls, while lean mass remained unaffected (Figure 4B). Additionally, iWAT, BAT, and liver weights were significantly lower in Adss1^AKO^ mice (Figure 4C). Consistent with their lean phenotype, Adss1^AKO^ mice exhibited decreased serum leptin (Figure 4D), improved glucose tolerance (Figure 4E,F), and insulin sensitivity (Figure 4G–H) compared to HFD‐fed controls. Furthermore, Adss1^AKO^ mice exhibited a significant increase in overall energy expenditure, VO_2,_ and VCO_2_ following β3‐adrenergic stimulation after adjustment for body weight, with a modest increase observed under basal conditions (Figure 4I–N; Figure S6B–D, Supporting Information). RER and physical activity remained comparable between the groups (Figure S6E–H, Supporting Information). These findings provide further evidence that Adss1^AKO^ mice possess an enhanced thermogenic capacity, even under metabolic stress conditions.

We next examined liver histology and found reduced lipid deposition in HFD‐fed Adss1^AKO^ mice (Figure 4O). Consistently, hepatic TG (Figure 4P), as well as serum AST (Figure 4Q), ALT (Figure 4R), and cholesterol (Figure 4S) levels, were all significantly lower in Adss1^AKO^ mice. Under conditions of nutrient overload, excessive NEFA can lead to ectopic lipid deposition.^[^ ^29^, ^30^ ^]^ Notably, we found that Adss1^AKO^ mice showed decreased serum levels of NEFA and glycerol (Figure 4T,U). To further investigate whether Adss1 knockdown could mitigate hepatic lipid deposition in obese *ob/ob* mice, we subcutaneously injected shAdss1‐ adeno‐associated virus (AAV) into the iWAT of 6‐week‐old *ob/ob* mice to induce Adss1 knockdown (Figure S6I, Supporting Information). This intervention led to significantly reduced hepatic TG levels (Figure S6J, Supporting Information), consistent with reduced histological lipid deposition in the liver (Figure S6K, Supporting Information). Additionally, serum glycerol levels (Figure S6L, Supporting Information) were significantly reduced, and NEFA levels (Figure S6M, Supporting Information) exhibited a downward trend in ob/ob mice with localized Adss1 knockdown. More importantly, the expression of thermogenic, fatty acid synthesis, and oxidation–related genes was markedly upregulated after Adss1 knockdown in iWAT of *ob/ob* mice (Figure S6N, Supporting Information).

Taken together, these findings indicate that Adss1 deletion enhances energy expenditure and protects against diet‐induced obesity. Furthermore, in both diet‐induced obese mice and *ob/ob* mice, Adss1 deletion alleviates hepatic lipotoxicity, at least in part by reducing serum NEFA levels.

### 2.5 Adss1 Depletion Upregulates Gk Expression and Facilitates Re‐Esterification Cycle in Adipose Tissue

**Figure 5:**
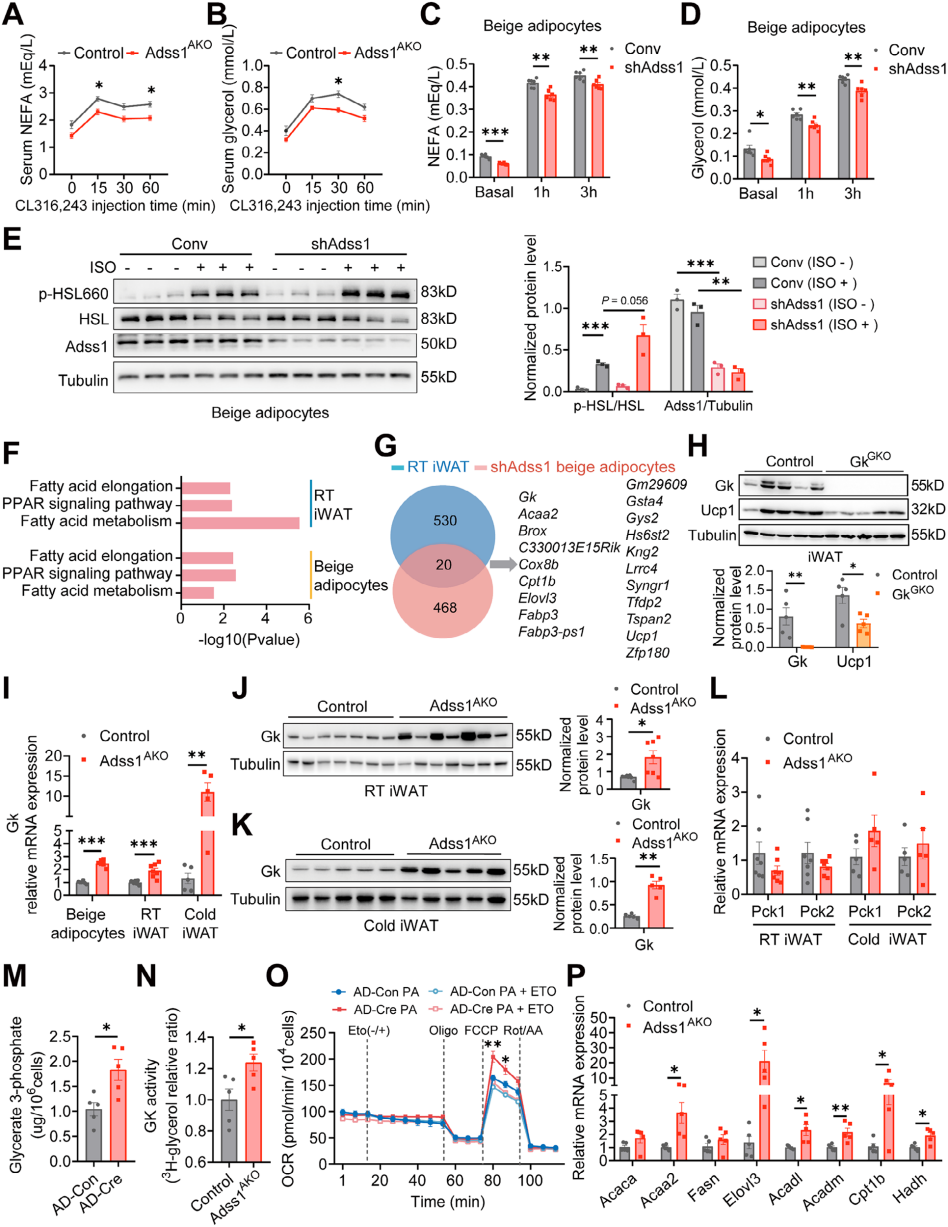
Gk‐mediated re‐esterification and lipid mobilization in Adss1‐deficient adipose tissue and beige adipocytes. A–B) Serum NEFA (A) and glycerol (B) levels in control and Adss1^AKO^ mice after treatment with 1 mg kg^−1^ CL316243 (*n* = 5). C–D) NEFA (C) and glycerol (D) concentrations in the supernatants of beige adipocytes infected with shAdss1 or control lentiviruses under basal and 5 µM ISO treatment (*n* = 6). E) Western blot analysis of p‐HSL in beige adipocytes treated with 5 µM ISO for 30 min (*n* = 3). F) Commonly upregulated KEGG pathways identified by RNA‐seq of iWAT in Adss1^AKO^ mice at RT and beige adipocytes with Adss1 knockdown (*n* = 4 and 3 per group for iWAT; *n* = 3 per group for beige adipocytes). G) Venn diagram showing commonly upregulated genes (Fold change > 1.5, *P* < 0.05). H) Western blot analysis and quantification of Gk and Ucp1 proteins in iWAT from 9‐week‐old CD‐fed control and Gk^AKO^ mice of iWAT after 3 days of cold exposure (*n* = 5). I) Gk mRNA levels in beige adipocytes (*n* = 6) and iWAT under different conditions (*n* = 7 for RT, *n* = 5 for cold exposure). J, K) Gk protein levels in iWAT from control and Adss1^AKO^ mice housed at RT (J, *n* = 7) or exposed to cold for 3 days (K, *n* = 5). L) mRNA levels of *Pck1* and *Pck2* in the iWAT from control and Adss1^AKO^ mice at RT (*n* = 7) and after 3 days of cold exposure (*n* = 5). M) Glycerate‐3‐phosphate levels in beige adipocytes with Adss1 deletion using Cre recombinase adenovirus (*n* = 5). N) Gk enzymatic activity in iWAT from control and Adss1^AKO^ mice after 3 days of cold exposure (*n* = 5). O) Fatty acid oxidation in beige adipocytes assessed by Seahorse extracellular flux analysis following Adss1 deletion via Cre recombinase adenovirus (*n* = 7). P) Expression of genes involved in fatty acid synthesis and oxidation in iWAT from control and Adss1^AKO^ mice after 3 days of cold exposure (*n* = 5). Data are presented as mean ± SEM. ^*^ *p* < 0.05, ^**^ *p* < 0.01, ^***^ *p* < 0.001. Two‐way ANOVA (A and B). Student's *t*‐test (C‐E, H‐P).

Beyond its role in adipose tissue energy regulation, Adss1 depletion also led to a significant reduction in serum NEFA and glycerol levels in Adss1^AKO^ mice under both cold exposure (Figure 2P,Q) and HFD conditions (Figure 4T,U), prompting our further investigation. To explore this, we further measured serum NEFA and glycerol levels following in vivo CL316243 stimulation. Notably, Adss1^AKO^ mice exhibited a downward trend in NEFA and glycerol levels prior to CL316243 injection, with a significant reduction upon stimulation (**Figure** **5**A,B). At the cellular level, Adss1 knockdown similarly decreased NEFA and glycerol concentrations in the supernatant under both basal and isoproterenol (ISO)‐stimulated conditions (Figure 5C,D). These findings raised the question of whether the reduced NEFA and glycerol release results from impaired lipolysis, enhanced re‐esterification, or increased fatty acid oxidation.

To address this, we first assessed lipolytic activity by measuring phosphorylated hormone‐sensitive lipase (p‐HSL) levels in beige adipocytes. Notably, Adss1‐deficient adipocytes exhibited increased p‐HSL under ISO stimulation (Figure 5E), indicating that the reduced NEFA and glycerol levels were not due to impaired lipolysis. To identify potential alternative mechanisms, we performed RNA sequencing on both iWAT from Adss1^AKO^ mice and primary Adss1‐knockdown beige adipocytes. Transcriptomic analysis revealed significant upregulation of fatty acid metabolism pathways in both experimental systems (Figure 5F). Through intersection analysis of the upregulated genes, we identified 20 common differentially expressed genes, including key lipid metabolism regulators such as *Gk*, *Cpt1b*, *Elovl3*, *Acaa2*, and *Fabp3* (Figure 5G). These findings suggest that the reduction in NEFA and glycerol release may be linked to enhanced re‐esterification or fatty acid oxidation. Notably, up to 50% of the NEFAs released during lipolysis are re‐esterified into triglycerides within adipocytes,^[^ ^10^, ^31^, ^32^ ^]^ highlighting the dynamic regulation of lipid flux. Gk is barely expressed in WAT, resulting in negligible formation of glycerol‐3‐phosphate (G3P) from glycerol. However, it is highly expressed in BAT and can be robustly induced by cold exposure in iWAT.^[^ ^33^, ^34^, ^35^ ^]^ Previous studies have indicated that AAV‐mediated knockdown of Gk in iWAT leads to a significant reduction in Ucp1 expression.^[^ ^35^ ^]^ Besides, Gk knockdown reduces p‐HSL levels in brown adipocytes.^[^ ^36^ ^]^ In line with these findings, we also observed a decrease in Ucp1 expression with Gk knockdown using siRNA in beige adipocytes (Figure S7A, Supporting Information), whereas Gk overexpression significantly enhanced Ucp1 levels (Figure S7B,C, Supporting Information). Additionally, we generated adipose‐specific Gk knockout (Gk^AKO^) mice (Figure S7D, Supporting Information) and found that, under 3 days of cold exposure, the thermogenic protein Ucp1 and key genes regulating thermogenesis and fatty acid metabolism were reduced in both iWAT (Figure 5H; Figure S7G, Supporting Information) and BAT (Figure S7H,I, Supporting Information). Moreover, thiazolidinedione (TZD) treatment has been reported to enhance Gk expression, facilitating re‐esterification and suppressing the release of NEFA and glycerol.^[^ ^14^ ^]^ Given the increased energy expenditure and reduced NEFA and glycerol secretion observed in Adss1^AKO^ mice, we hypothesize that Gk functions as a key downstream effector mediating the metabolic effects of Adss1.

We further demonstrated that Adss1 deletion significantly upregulates Gk expression in beige adipocytes (Figure 5I). Specially, Gk expression was markedly elevated in iWAT of Adss1^AKO^ mice under both RT and cold‐stimulated conditions (Figure 5I–K), whereas other re‐esterification‐related genes, such as *Pck1* and *Pck2*, remained unchanged (Figure 5L). Similarly, in human adipocytes, siRNA‐mediated knockdown of ADSS1 also resulted in a significant upregulation of the thermogenic protein UCP1 and GK (Figure S7J, Supporting Information). Consistent with this, Adss1 knockdown in beige adipocytes led to an increase in G3P levels (Figure 5M). More importantly, isotopic tracing using ^3^H‐glycerol revealed a higher radioactivity signal in the iWAT of Adss1^AKO^ mice (Figure 5N), confirming that Gk‐mediated glycerol re‐esterification was indeed enhanced. Beyond re‐esterification, we assessed fatty acid oxidation and found an increased capacity for palmitic acid oxidation after Adss1 knockdown in beige adipocytes, as determined by Seahorse analysis (Figure 5O). Moreover, qPCR analysis revealed the upregulation of key genes involved in fatty acid synthesis and oxidation in iWAT of Adss1^AKO^ mice, including *Acaa2*, *Elovl3*, *Acadl*, *Acadm*, *Cpt1b*, and *Hadh* (Figure 5P). These findings suggest that Adss1 deletion enhances lipid cycling by promoting both glycerol re‐esterification and subsequent lipid oxidation and synthesis.

Interestingly, serum NEFA (Figure S8A, Supporting Information) and glycerol (Figure S8B, Supporting Information) levels were significantly and consistently reduced in aging Adss1^AKO^ mice compared to littermate controls. Concurrently, Gk mRNA expression was markedly upregulated in these mice (Figure S8C, Supporting Information). Given that NEFAs serve as the primary energy source for cardiomyocytes,^[^ ^37^, ^38^ ^]^ we assessed cardiac function in aging mice. Unexpectedly, while diastolic function remained unaffected (Figure S8E,F, Supporting Information), cardiac contractile function was impaired in aging Adss1^AKO^ mice (Figure S8G,H, Supporting Information). Consistently, the expression of genes involved in fatty acid oxidation, such as *Cpt1b* and *Acadm*, was reduced in the myocardium of Adss1^AKO^ mice, suggesting that impaired cardiac contractile function in these mice may be attributed to limited fatty acid availability for energy production (Figure S8I, Supporting Information).

These findings suggest that while Gk‐mediated metabolic remodeling enhances energy expenditure and lipid homeostasis in young Adss1^AKO^ mice, prolonged suppression of circulating NEFA in aging mice may impair cardiac function, highlighting the complex physiological consequences of sustained adipose tissue metabolic adaptation.

### 2.6 Gk is Indispensable for the Function of Adss1 in Fat

**Figure 6:**
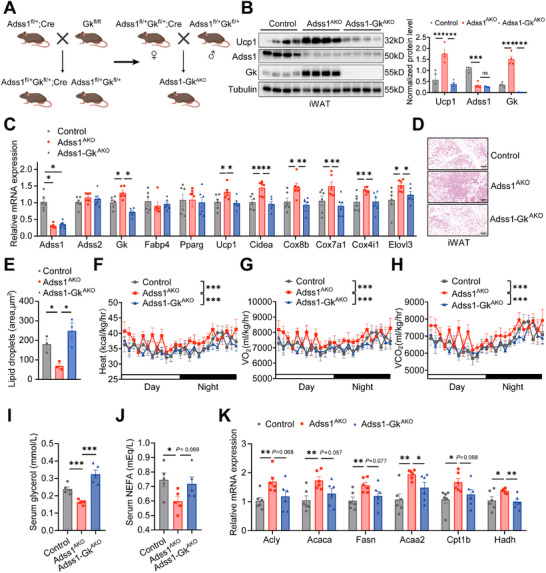
Essential role of Gk in Adss1‐mediated adipose tissue function. A) Schematic representation of the adipose tissue‐specific Adss1‐Gk^AKO^ mouse model. B–E) Metabolic parameters were assessed in littermate control, Adss1^AKO,^ and Adss1‐Gk^AKO^ mice after 3 days of cold exposure. Protein levels and quantification in iWAT (*n* = 4) (B). mRNA expression of Adss1, Adss2, Gk, adipocyte marker genes, and thermogenic genes in iWAT (*n* = 6) (C). Representative H&E staining (D) and quantification of lipid droplet area of iWAT (E), scale bar=100 µm. F–H) Metabolic cage analysis after 3 days cold exposure, including heat production (F), VO_2_ (G)_,_ and VCO_2_ (H) (*n* = 5). I–J) Serum glycerol (I) and NEFA (J) concentrations following 3 days of cold exposure (*n* = 5). K) Expression levels of key genes in fatty acid synthesis and oxidation in iWAT (*n* = 6). Data are presented as mean ± SEM. ^*^ *p* < 0.05, ^**^ *p* < 0.01, ^***^ *p* < 0.001. Student's *t*‐test (B, C, E, I‐K). Two‐way ANOVA (F‐H).

To further investigate whether the metabolic phenotypes observed in Adss1^AKO^ mice were driven by the upregulation of Gk expression, we generated adipose tissue‐specific Adss1 and Gk double knockout mice (Adss1‐Gk^AKO^) by crossing Adss1 heterozygous mice with Gk floxed mice (**Figure** **6**A,B; Figure S9A–D, Supporting Information). Under RT conditions, we found that thermogenesis‐related genes were significantly upregulated in iWAT of Adss1^AKO^ mice, which was reversed in Adss1‐Gk^AKO^ mice (Figure S9E, Supporting Information). Similarly, the elevated expression of the thermogenic protein Ucp1 observed in iWAT of Adss1^AKO^ mice was also abolished in Adss1‐Gk^AKO^ mice (Figure S9F, Supporting Information). Consistent with our previous results in Adss1^AKO^ mice, the expression of the thermogenic protein Ucp1 in BAT remained unchanged in both Adss1^AKO^ and Adss1‐Gk^AKO^ mice (Figure S9G, Supporting Information). At 10 weeks of age, Adss1‐Gk^AKO^ mice, along with their littermate controls and Adss1^AKO^ mice, were subjected to cold exposure to assess thermogenic function. Our findings demonstrated that the upregulation of Ucp1 protein level (Figure 6B), as well as the increased expression of thermogenic genes such as *Ucp1*, *Cidea*, *Cox8b*, and *Cox7a1*, observed in Adss1^AKO^ mice, was abolished in Adss1‐Gk^AKO^ mice (Figure 6C). Moreover, the morphological browning of iWAT in Adss1^AKO^ mice was markedly attenuated in Adss1‐Gk^AKO^ mice, indicating a critical role of Gk in this process (Figure 6D‐E). Consistently, Adss1‐Gk^AKO^ mice reversed the enhanced heat production, VO_2,_ and VCO_2_ in Adss1^AKO^ mice (Figure 6F–H), with no significant differences in RER (Figure S9H, Supporting Information). Importantly, the deletion of Gk in adipose tissue effectively prevented the reductions in circulating NEFA and glycerol levels that were observed in Adss1^AKO^ mice (Figure 6I,J). In line with these metabolic changes, the upregulated genes involved in fatty acid synthesis (*Acaca*, *Fasn*) and oxidation (*Acaa2*, *Cpt1b*, *Hadh*) in Adss1^AKO^ mice were significantly attenuated in Adss1‐Gk^AKO^ mice (Figure 6K).

To determine whether targeted Gk inhibition in iWAT can reverse the metabolic alterations in Adss1^AKO^ mice, we locally injected AAV‐Gk into the iWAT of both control and Adss1^AKO^ mice to reduce Gk expression. Four weeks post‐injection, mice were housed individually under cold conditions for three days. As expected, Gk knockdown effectively reversed circulating NEFA and glycerol levels in Adss1^AKO^ mice (Figure S9I,J, Supporting Information) and abolished the upregulation of Ucp1 protein (Figure S9K, Supporting Information). These findings confirm that Gk depletion in iWAT is sufficient to modulate the phenotypes in Adss1^AKO^ mice. Collectively, our results establish Gk as a critical downstream effector of Adss1, driving its regulatory influence on lipid cycling and thermogenic adaptation in adipose tissue.

### 2.7 Adss1 Deletion Selectively Remodels Histone Acetylation of Gk

**Figure 7:**
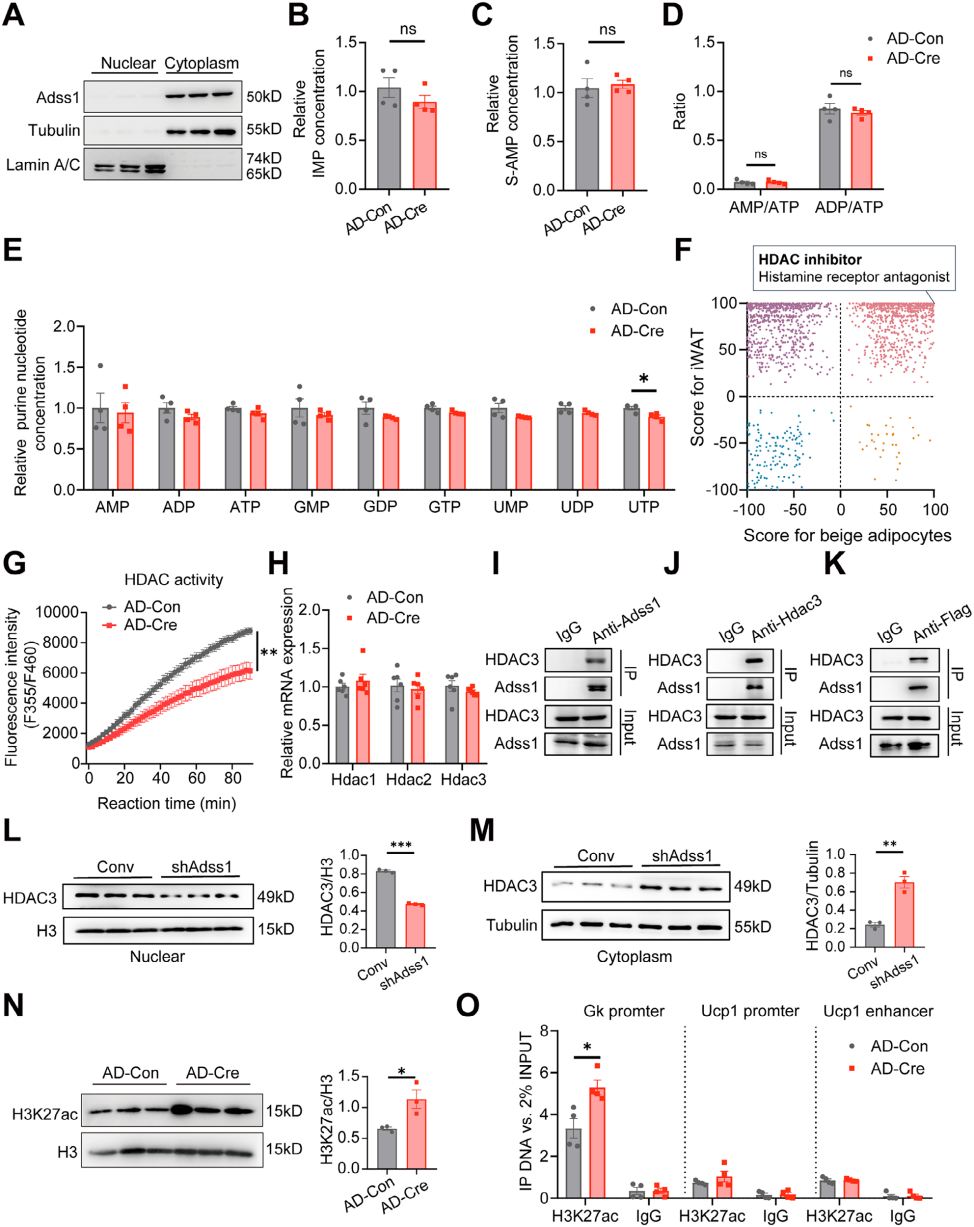
Adss1‐mediated regulation of Gk expression depends on histone acetylation. A) Western blot analysis of Adss1 expression in nuclear and cytoplasmic fractions of adipocytes. B–E) Targeted metabolomics analysis of purine nucleotide levels in beige adipocytes with Adss1 knockdown via Cre recombinase adenovirus (*n* = 4), including IMP (B), S‐AMP (C), AMP/ATP, and ADP/ATP ratio (D), and other purine nucleotides (E). F) 2D plot of enrichment scores derived from CMap analysis comparing iWAT from control and Adss1^AKO^ mice at RT and beige adipocytes with Adss1 knockdown. Each point represents a small molecule in the selected cell line. G) HDAC activity in Adss1‐deficient beige adipocytes (*n* = 6). H) mRNA expression of the indicated genes in beige adipocytes with Adss1 knockdown (*n* = 6). I) Endogenous Co‐IP with anti‐Adss1 antibody in beige adipocytes. J) Endogenous Co‐IP with anti‐HDAC3 antibody in beige adipocytes. K) Semi‐endogenous Co‐IP showing overexpressed Flag‐Adss1 interacts with endogenous HDAC3, with IgG as a negative control. L) Nuclear HDAC3 protein levels in Adss1 knockdown beige adipocytes (*n* = 3). M) Cytoplasmic HDAC protein levels in Adss1 knockdown beige adipocytes (*n* = 3). N) H3K27ac protein levels in Adss1 knockdown beige adipocytes (*n* = 3). O) H3K27ac chromatin immunoprecipitation followed by ChIP‐qPCR analysis at the Gk locus in Adss1‐deficient beige adipocytes (*n* = 4). Data are presented as mean ± SEM. ^*^ *p* < 0.05, ^**^ *p* < 0.01. Student's *t*‐test (B‐E, H, L‐O). Two‐way ANOVA was performed for G.

We found that Adss1 protein in adipose tissue is localized in the cytoplasm (**Figure** **7**A), consistent with previous studies.^[^ ^22^ ^]^ Given this cytoplasmic localization, we investigated whether Adss1 influences Gk expression by modulating cytoplasmic purine nucleotide concentrations. Previous study has shown that IMP plays a role in regulating thermogenic gene expression and promoting iWAT browning.^[^ ^39^ ^]^ Since IMP is a direct upstream product of Adss1, we hypothesized that Adss1 knockdown might lead to its accumulation. To test this, we quantified purine nucleotide levels in beige adipocytes lacking Adss1. However, targeted metabolomic analysis revealed no significant changes in IMP levels (Figure 7B), or its downstream product, S‐AMP (Figure 7C). Purine nucleotides, particularly the AMP/ATP and ADP/ATP ratios, are known to regulate energy metabolism via the AMPK pathway.^[^ ^40^, ^41^, ^42^ ^]^ Our analysis showed no statistically significant changes in the AMP/ATP or ADP/ATP ratios, nor in individual nucleotide levels (Figure 7D,E). Moreover, we utilized Cre recombinase to deplete AMPK in beige adipocytes derived from AMPK‐floxed mice, in combination with lentivirus‐mediated Adss1 knockdown. Notably, the increase in Ucp1 expression observed following Adss1 silencing persisted even in the absence of AMPK (Figure S10A, Supporting Information), indicating that the functional alterations in adipocytes mediated by Adss1 occur independently of the AMPK pathway.

To further elucidate the mechanism of Adss1‐mediated effects, we queried the CMap database with the top 150 upregulated genes from both the iWAT of Adss1^AKO^ mice and Adss1‐deficient beige adipocytes. Compounds were ranked based on their enrichment scores and visualized in a 2D plot. Intriguingly, compounds located in the first quadrant, marked by red points, demonstrated positive enrichment in both datasets, suggesting functional similarities to Adss1 deficiency. Among these, the HDAC inhibitor (HDAC3 selective) and histamine receptor antagonist achieved exceptionally high scores (>99), indicating strong concordance with the transcriptional profile induced by Adss1 deficiency (Figure 7F). This is particularly notable given the well‐established role of HDACs in adipose tissue biology. HDACs regulate gene expression through histone acetylation, and specifically inhibiting HDAC can enhance thermogenic function in adipose tissue.^[^ ^43^, ^44^, ^45^ ^]^ Notably, adipose‐specific deletion of HDAC3 has been reported to induce iWAT browning and enhance the futile lipid cycle,^[^ ^46^ ^]^ further supporting the potential functional link between HDAC inhibition and the observed effects of Adss1 deficiency. Therefore, we first examined HDAC activity in Adss1‐deficient beige adipocytes. As expected, we observed a significant reduction in HDAC activity (Figure 7G). Meanwhile, there were no changes in *Hdac1*, *Hdac2*, and *Hdac3* mRNA expression (Figure 7H). Subsequently, we performed targeted metabolomic profiling to assess intracellular levels of metabolic intermediates reported to regulate HDAC activity, including acetyl‐CoA, CoA, butyric acid, and β‐hydroxybutyric acid, and found no significant differences between groups (Figure S10B, Supporting Information). To further elucidate the mechanism by which Adss1 deficiency reduces HDAC activity, we investigated the functional interaction between Adss1 and HDAC. In beige adipocytes, endogenous Adss1 was found to interact with HDAC3 (Figure 7I,J), and a similar association was confirmed when Flag‐Adss1 was overexpressed (Figure 7K). Notably, loss of Adss1 altered the subcellular distribution of HDAC3, resulting in decreased nuclear accumulation (Figure 7L) and concomitant cytoplasmic retention (Figure 7M). These findings point to a role of Adss1 in controlling HDAC3 dynamics and suggest potential consequences for histone acetylation.

H3K27 acetylation is a critical histone mark for thermogenic gene activation,^[^ ^47^ ^]^so we examined its level in Adss1‐deficient beige adipocytes. Acid‐extracted histones revealed that Adss1 deficiency significantly increased H3K27ac in beige adipocytes compared to controls (Figure 7N). In contrast, other histone modifications—including H3K9ac, H3K4me3, and H3K27me3 showed no significant change (Figure S10C–E, Supporting Information). These findings indicate that Adss1 loss selectively elevates H3K27ac, potentially facilitating a thermogenic transcriptional program. Consistent with this notion, analysis of published H3K27ac ChIP‐seq data (GSE108077) ^[^ ^48^ ^]^ revealed that cold exposure can markedly enhance H3K27ac enrichment at the Gk promoter in iWAT (Figure S10F, Supporting Information). This suggests an epigenetic mechanism whereby increased H3K27ac at the Gk locus under cold conditions promotes Gk transcriptional activation, contributing to adipose tissue remodeling during browning. Importantly, chromatin immunoprecipitation (ChIP) analysis using an H3K27ac antibody confirmed the increased H3K27 acetylation at the Gk promoter in Adss1‐knockdown mature beige adipocytes, whereas H3K27 acetylation levels at the Ucp1 promoter and enhancer regions remained unchanged (Figure 7O). These results indicate that Adss1 deficiency selectively enhances H3K27 acetylation at the Gk promoter, providing a mechanistic link between Adss1 and Gk transcriptional regulation.

### 2.8 Human Adipose Tissue ADSS1 is Correlated with Metabolic Traits

**Figure 8:**
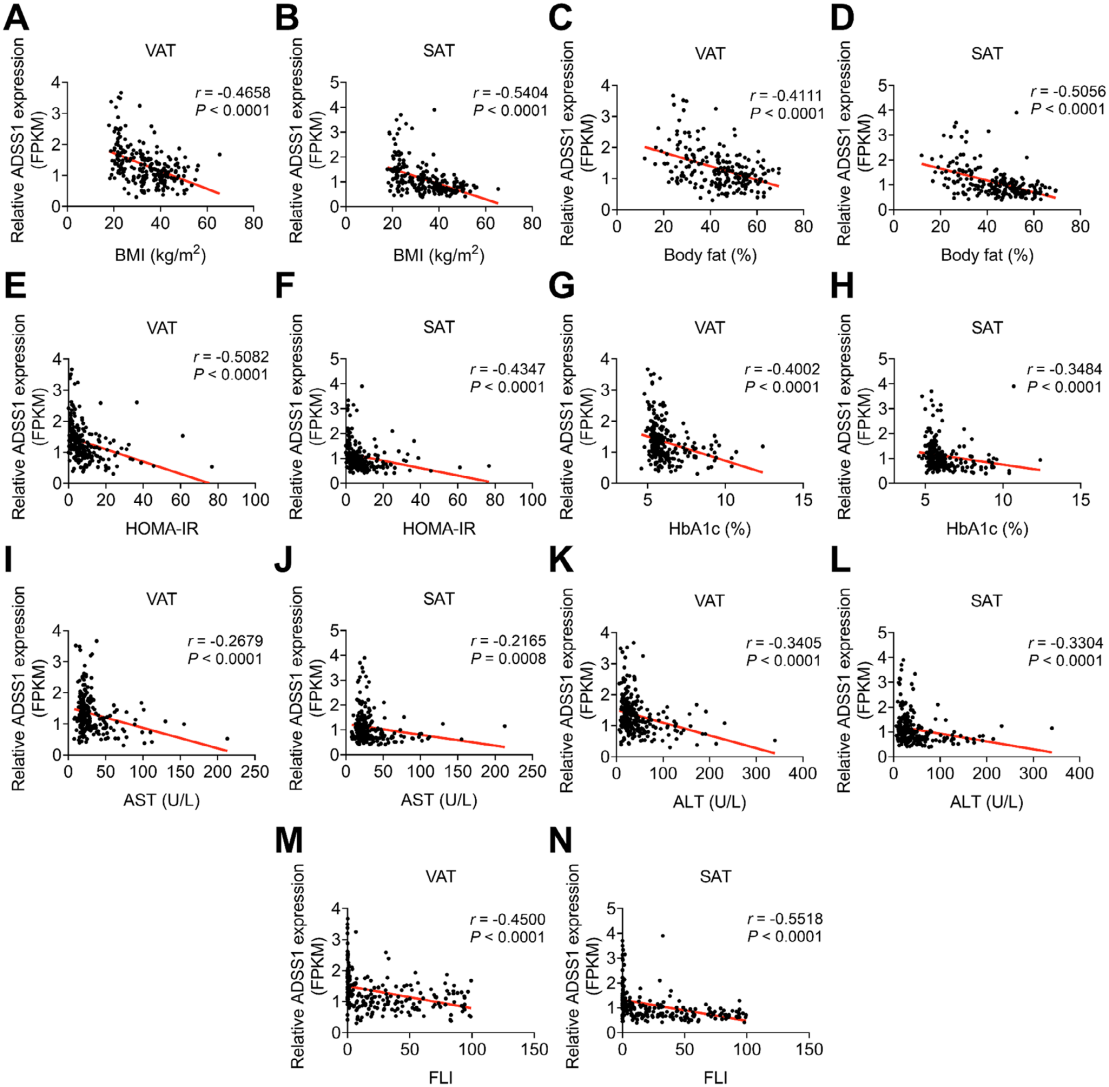
ADSS1 expression in human adipose tissue correlates with obesity‐associated metabolic traits. A–N) Spearman's correlation between ADSS1 expression and key metabolic parameters in VAT and SAT, including BMI (A,B, *n* = 236), body fat percentage (C,D, *n* = 217), HOMA‐IR (E,F, *n* = 215), HbA1c (G,H, *n* = 232), AST (I,J, *n* = 236), ALT (K,L, *n* = 236), and FLI (M,N, *n* = 227). Correlation coefficients (*r*) and two‐tailed *P* values are shown. FPKM: fragments per kilobase of transcript per million mapped reads.

To investigate the clinical relevance of ADSS1 in obesity, we analyzed adipose tissue samples from lean, overweight, and obese individuals. ADSS1 mRNA expression in both VAT and SAT exhibited a significant inverse correlation with BMI and body fat percentage (**Figure** **8**A–D), whereas no significant correlation was observed for ADSS2 (Figure S11A–D, Supporting Information). Notably, ADSS1 expression in VAT and SAT was also inversely associated with HOMA‐IR and HbA1c (Figure 8E–H). Moreover, ADSS1 expression was negatively correlated with liver disease markers, including AST and ALT (Figure 8I–L). The FLI, a key indicator of hepatic lipid deposition, was also inversely correlated with ADSS1 expression in both VAT and SAT (Figure 8M,N). In contrast, ADSS2 showed no significant correlation with these clinical parameters (Figure S11E–N, Supporting Information). Collectively, these findings strongly suggest that, in human adipose tissue, ADSS1 may play a causal role in the pathogenesis of obesity and its associated metabolic dysfunctions.

## 3 Discussion

**Figure 9:**
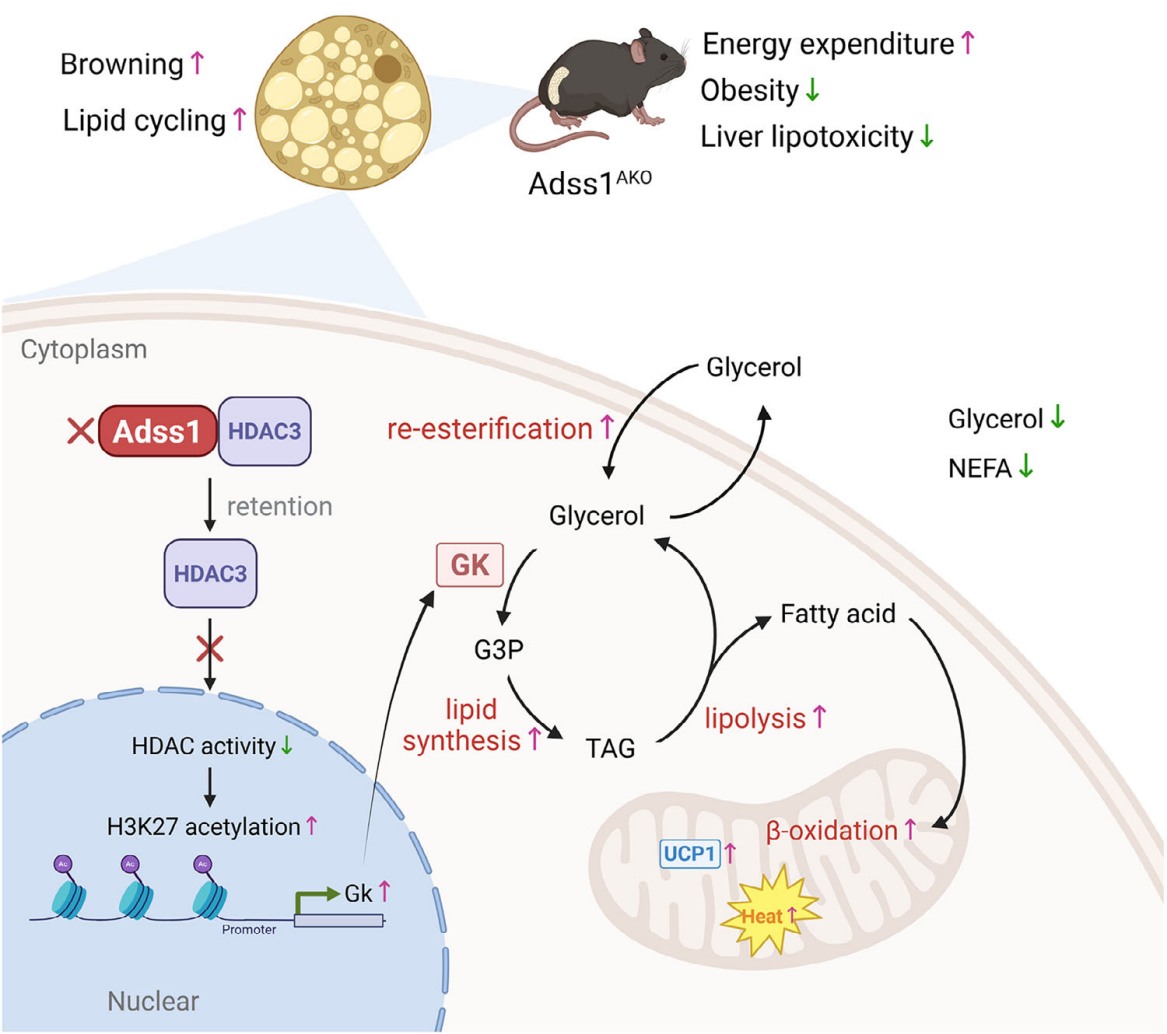
Working model of Adss1‐mediated regulation of energy metabolism in adipose tissue. In beige adipocytes, Adss1 interacts with HDAC3 in the cytoplasm, and its loss reduces nuclear HDAC3 while increasing cytosolic fractions. This redistribution suppresses HDAC activity and enhances H3K27 acetylation at the Gk promoter, leading to transcriptional activation of Gk. Elevated Gk expression promotes glycerol‐dependent fatty acid re‐esterification, initiating a lipid synthesis–oxidation cycle that drives browning and thermogenesis. Consequently, Adss1^AKO^ mice show increased energy expenditure and are protected against diet‐induced obesity and hepatic lipotoxicity. Created in BioRender. Sun, J. (2025). https://BioRender.com/24m9oz2.

In this study, we reveal a previously unrecognized role of Adss1 in regulating adipose tissue homeostasis, broadening our insight into the contribution of purine nucleotide synthesis enzymes to energy metabolism. Our findings reveal that Adss1^AKO^ mice exhibit pronounced iWAT browning under RT conditions. Upon cold exposure, Adss1^AKO^ mice display increased energy expenditure, enabling to maintain core body temperature more efficiently. Additionally, these mice demonstrate resistance to diet‐induced obesity, along with improved glucose tolerance, enhanced insulin sensitivity, and reduced hepatic lipid accumulation (**Figure** **9**).

Previous studies have largely underestimated the role of purine nucleotide synthases in fat thermogenesis, mainly due to the minimal changes observed in BAT following cold stimulation. However, our study revealed a robust upregulation of Adss1 in iWAT in response to cold exposure, β‐adrenergic stimulation, and physical exercise, suggesting a previously unrecognized role of Adss1 and adaptive thermogenic responses. This expression pattern is reminiscent of other genes, such as Pwwp2b,^[^ ^49^ ^]^ Kcnk3,^[^ ^50^ ^]^ and 5′‐truncated adenylyl cyclase 3 mRNA isoform (Adcy3‐at) ^[^ ^51^ ^]^ reported to be significantly upregulated in adipose tissue under similar conditions. However, these genes function as negative regulators of thermogenesis, and their deletion enhances the thermogenic program in adipose tissue, consistent with our findings. These rheostat molecules act as physiological brakes, preventing excessive thermogenesis and preserving systemic energy balance.^[^ ^51^ ^]^ Thus, we propose that Adss1 functions as a negative regulator of thermogenesis, limiting excessive activation in adipose tissue to prevent unnecessary energy expenditure.

Further investigation revealed that Gk is a key downstream effector of Adss1 ablation, driving re‐esterification and subsequently promoting adipose tissue browning and energy metabolism. Gk catalyzes the conversion of glycerol to G3P, a key substrate for TG synthesis.^[^ ^52^ ^]^ The futile re‐esterification cycle depends on a sufficient G3P supply to sustain TG formation.^[^ ^10^, ^53^ ^]^ Typically, due to the low glycerol kinase (Gk) activity in white adipose tissue, glyceroneogenesis, mediated by phosphoenolpyruvate carboxykinase (PEPCK‐C), serves as the primary source of G3P.^[^ ^31^, ^54^ ^]^ However, we observed no significant changes in Pck expression. Instead, Gk expression was robustly upregulated following Adss1 deletion, both in vitro and in vivo, under RT conditions (in both young and aged mice) as well as cold exposure. Guan et al. reported that TZDs upregulate Gk expression in adipocytes, promoting glycerol incorporation into TG and reducing NEFA release through futile re‐esterification.^[^ ^14^ ^]^ Our study identified an additional upstream regulator of Gk. The deletion of Adss1 led to increased Gk expression, which in turn facilitated Gk‐mediated glycerol re‐esterification, as demonstrated by the enhanced incorporation of ^3^H‐glycerol into adipocytes. Moreover, this re‐esterification process significantly reduced the release of NEFA and glycerol into the bloodstream, thereby potentially alleviating lipotoxic effects on the liver during nutrient excess conditions.

Furthermore, our study revealed that Adss1 deficiency‐induced re‐esterification is accompanied by enhanced lipid synthesis and oxidation cycling in iWAT. This metabolic signature parallels the processes observed in active BAT, where concurrent upregulation of lipid synthesis and oxidation genes has been documented and extensively studied.^[^ ^55^, ^56^, ^57^, ^58^ ^]^ Supporting this observation, Gurmaches et al. demonstrated that AKT2 promotes de novo lipogenesis in adipocytes by activating ChREBPβ transcriptional activity, thereby generating additional fuel sources to support both energy storage and thermogenic processes.^[^ ^57^ ^]^ These findings collectively suggest that Adss1 knockout‐mediated enhancement of lipid synthesis and oxidation cycling, potentially providing adequate fuel turnover to support the increased metabolic requirements during thermogenic activation. Additionally, Iwase et al. reported that Gk stimulates Ucp1 expression, potentially through mechanisms involving intracellular monounsaturated fatty acid levels.^[^ ^35^ ^]^ Given that lipid cycling enhances lipid diversity,^[^ ^11^, ^59^ ^]^ further studies are needed to determine whether Adss1 deficiency‐induced Gk upregulation influences thermogenic gene transcription by modulating lipid composition.

Although nucleotide metabolism is broadly linked to cellular energy status, our study found no significant changes in purine nucleotide levels or AMPK activity. It has been reported that the IMP–S‐AMP–AMP cycle plays a central role in energy metabolism, exerting direct and immediate influence on glycolysis, electron transport, fatty acid oxidation, and the TCA cycle.^[^ ^60^ ^]^ In Drosophila, heterozygous mutations in the Adss gene (encoding the only ADSS enzyme in flies) elevate AMP/ATP and ADP/ATP ratios, leading to increased AMPK phosphorylation and potentially contributing to lifespan extension.^[^ ^61^ ^]^ However, our study demonstrated that Adss1 regulates energy metabolism independently of AMPK, possibly due to functional differences among isoenzymes across different species. In contrast, we observed a sustained reduction of serum NEFA in aging Adss1^AKO^ mice, which led to impaired cardiac contractile function. These findings suggest that while Adss1 deficiency may protect against hepatic lipotoxicity under nutrient‐overload conditions, its prolonged effects on circulating NEFA levels could have detrimental consequences for organs such as the heart, which rely primarily on fatty acids for energy.

Metabolic enzymes and their respective metabolites are vital for chromatin structure and transcriptional responses, exerting their effects through various epigenetic mechanisms, including histone modifications, chromatin remodeling, and DNA methylation.^[^ ^62^, ^63^, ^64^ ^]^ Consistent with these findings, a recent study demonstrated that disruption of de novo synthesis or purine uptake—achieved through the knockout of genes involved in purine metabolism—specifically impaired the functionality of BRD4, a key reader of lysine acetylation, thereby establishing a direct connection between nucleotide metabolism and epigenetic regulation.^[^ ^64^ ^]^ In addition, several studies have shown that many metabolic enzymes, previously considered to function exclusively in the cytoplasm, can also localize to the nucleus, where they moonlight in regulating chromatin structure and gene expression independently of their catalytic activity. For instance, the nucleotide biosynthetic enzyme guanosine monophosphate synthase (GMPS) has been shown to interact with USP7, thus facilitating histone H2B deubiquitination and promoting chromatin silencing, thereby modulating target gene expression.

In this study, we observed that silencing Adss1 in adipocytes reduced HDAC activity and selectively increased H3K27 acetylation at the Gk promoter, implicating Adss1 in the epigenetic regulation of the re‐esterification cycle of adipocytes. We first examined the subcellular localization of Adss1 and confirmed that it is exclusively expressed in the cytoplasm, with no detectable nuclear presence. We then measured intracellular purine nucleotide levels and found no significant changes in either individual nucleotides or the total nucleotide pool upon Adss1 knockdown, indicating that the reduction in HDAC activity is not mediated by alterations in purine metabolite abundance. Because HDAC3 enzymatic activity is also coupled to its nuclear localization, with cytoplasmic retention attenuating its deacetylase function,^[^ ^66^ ^]^ we therefore examined whether Adss1 influences HDAC3 distribution. To address this possibility, we tested for direct protein interactions and found that Adss1 physically associates with HDAC3 in beige adipocytes. Loss of Adss1 disrupted HDAC3 nucleocytoplasmic distribution, leading to reduced nuclear accumulation and increased cytoplasmic retention. This redistribution provides a compelling explanation for the diminished nuclear HDAC activity observed in Adss1‐deficient cells. Collectively, these findings reveal that Adss1, beyond its canonical metabolic role, acts as a cytoplasmic regulator of HDAC3 localization and activity, thereby linking a purine biosynthetic enzyme to epigenetic control of adipocyte remodeling and thermogenic activation. Given that HDAC3 nucleocytoplasmic shuttling is governed by multiple intricate mechanisms—including phosphorylation‐dependent interactions with 14‐3‐3 proteins,^[^ ^67^ ^]^ regulation by importin/exportin systems,^[^ ^68^ ^]^ modulation through signaling kinases and phosphatases,^[^ ^69^ ^]^ and interactions with cofactors or chaperone proteins that regulate complex stability and subcellular localization,^[^ ^70^ ^]^ a comprehensive mechanistic dissection remains to be further explored.

In summary, our study identifies Adss1 as a crucial regulator of adipose tissue function. Its deficiency reduces HDAC activity and thereby facilitates epigenetic upregulation of Gk and enhanced glycerol‐dependent fatty acid re‐esterification cycle. This adaptation promotes iWAT browning, alleviates lipotoxicity, and contributes to systemic metabolic homeostasis. These findings may represent a potential strategy for therapeutic intervention in obesity and related metabolic disorders.

## 4 Limitations of the Study

This study has several limitations. First, although steady‐state purine nucleotide levels were unchanged, we did not assess metabolic flux, and dynamic alterations in purine turnover remain a plausible but unproven mechanism. Second, although our experiments established an interaction between Adss1 and HDAC3 and demonstrated altered HDAC3 nuclear–cytoplasmic distribution upon Adss1 loss, the precise mechanism by which this interaction regulates HDAC3 localization remains to be clarified. Third, although RNA‐seq and ChIP–qPCR provide initial evidence and our CMap–ChIP approach is well supported, additional orthogonal methods—such as CUT&Tag, ATAC‐seq, CRISPR‐based epigenetic editing, or proteomics—will be required to rigorously validate and strengthen this mechanistic link.

## 5 Experimental Section

### Animal Models and Husbandry

Adss1^fl/fl^ mice were generated from a C57BL/6J background by introducing loxP sites flanking exon 2. Adipose tissue‐specific Adss1 knockout mice (Adss1^AKO^) were obtained by crossing Adss1^fl/fl^ mice with Adiponectin‐Cre mice, which were kindly provided by Dr. Xinran Ma's laboratory at East China Normal University. Gk^fl/fl^ mice were purchased from GemPharmatech Co., Ltd. (Nanjing, China) and were generated from C57BL/6J embryonic stem cells with LoxP sites flanking exons 2–3 of the Gk gene. Adipose tissue‐specific Gk knockout mice (Gk^AKO^) were obtained by crossing Gk^fl/fl^ mice with Adiponectin‐Cre mice. To generate adipose tissue‐specific Adss1‐Gk double knockout mice (Adss1‐Gk^AKO^), Adss1^fl/+^; Cre mice were first crossed with Gk^fl/fl^ mice to produce Adss1^fl/+^ Gk^fl/+^; Cre and Adss1^fl/+^ Gk^fl/+^ offspring. Considering that the Gk is located on the X chromosome, female Adss1^fl/+^ Gk^fl/+^; Cre mice were subsequently bred with male Adss1^fl/+^ Gk^fl/+^ mice to obtain double knockout progeny. AMPK^fl/fl^ mice were kindly provided by Dr. Chensong Zhang's laboratory at Xiamen University. Genotyping of the offspring was performed by PCR analysis of genomic DNA extracted from tail tissues. The primers used are listed in Table S1 (Supporting Information).

Mice were housed in a specific pathogen‐free (SPF) environment, at RT (22–24 °C) with a relative humidity of 50–60%, maintained under a 12‐h light/12‐h dark cycle. Age‐matched male littermate mice were used for all experiments. Unless otherwise specified, mice had ad libitum access to water and chow diet (CD). For high‐fat diet (HFD) challenge experiments, male C57BL/6J mice were fed a 60% fat (Research Diets, D12492) starting at 8–10 weeks of age. For cold exposure experiments, mice were housed individually with unrestricted access to water and food throughout the exposure period. All animal experiments were approved by the Animal Care Committee of Shanghai Sixth People's Hospital, affiliated to Shanghai Jiao Tong University School of Medicine.

### Isolation and Differentiation of Stromal Vascular Fraction Cells

The stromal vascular fractions were isolated from the iWAT and BAT pads of 6–8‐week‐old WT, Adss1^fl/fl^, and Adss1^AKO^ mice, following to previously described protocols.^[^ ^71^, ^72^ ^]^ Briefly, dissected adipose tissue was finely minced and digested in Dulbecco's modified Eagle medium (DMEM) containing 0.2% collagenase (Sigma, C6885) and 1.5% bovine serum albumin (BSA; Solarbio, A8020) at 37 °C for 40–60 min. The digested tissue was then filtered through 40 µm cell strainers (Falcon, 352340) and centrifuged to pellet the stromal vascular cell (SVC). The SVC pellet was resuspended in DMEM supplemented with 10% fetal bovine serum (FBS, Gibco) and seeded into culture plates. Cells were maintained at 37 °C in a humidified incubator with 5% CO_2_. Upon reaching confluence, differentiation into beige or brown adipocytes was induced using a differentiation medium consisting of DMEM supplemented with 10% FBS, 1% penicillin/streptomycin, 0.5 mM 3‐isobutyl‐1‐methylxanthine (IBMX, Sigma, I7018), 1 µM insulin (Lily, HI0240), 5 µM rosiglitazone (Sigma, R2408), 1 µM dexamethasone (Sigma, D4902), and 50 nM T3 (Sigma, T2877). After 2 days of induction, cells were switched to a maintenance medium containing 10% FBS, 5 µM rosiglitazone, 50 nM T3, and 1 µM insulin, and cultured for an additional 4–6 days to allow full differentiation. For the isolation and differentiation of human adipocytes, stromal vascular fraction (SVF) cells were obtained from human adipose tissue through collagenase II digestion, as previously described.^[^ ^73^ ^]^ Once the SVF cells reached confluence, adipogenic differentiation was induced using an induction medium containing 0.5 mM IBMX, 1 µM insulin, 1 µM dexamethasone, 0.5 µM T3, and 10 µM rosiglitazone for 4 days. Subsequently, the medium was replaced with a maintenance medium composed of 10 µM rosiglitazone, 0.5 µM T3, and 1 µM insulin, and the cells were further cultured for an additional 17 days to achieve full differentiation.

### Cold Exposure and Swimming Exercise Studies

To assess the thermogenic function of the iWAT and BAT, 6–8‐week‐old mice were exposed to 4–5 °C for either 3 or 7 days. To monitor body temperature, mice were first acclimated under thermoneutral conditions (30 °C) for 2 weeks, and then subjected to cold exposure at 5 °C. Rectal temperature was recorded at 0, 2, 4, 6, and 8 h using a rectal probe. During all cold exposure experiments, mice were singly housed with ad libitum access to food and water.

The swimming exercise protocol was adapted from a previously described method with minor modifications.^[^ ^74^ ^]^ Briefly, the exercise regimen began at 8 weeks of age and included two phases: an acclimatization phase followed by a training phase. During the acclimatization phase, mice underwent 10‐min swim sessions twice daily, with the duration increasing by 10 min per day until reaching 90 min per session over the course of 9 days. The subsequent training phase consisted of 90‐min swim sessions, also performed twice daily, for an additional 14 days.

### Clinical Acquisition of Human Adipose Tissue for RNA‐seq

Human adipose tissue samples were obtained from individuals undergoing elective abdominal cholecystectomy or bariatric surgery at Shanghai Sixth People's Hospital Affiliated to Shanghai Jiao Tong University School of Medicine. A total of 236 participants were included in the study, comprising 87 individuals with normal weight and 149 individuals with obesity. Detailed information regarding participant recruitment, exclusion criteria, and clinical characteristics has been described in the previously published studies.^[^ ^75^ ^]^ The human study was approved by the Ethics Committee of Shanghai Sixth People's Hospital Affiliated to Shanghai Jiao Tong University School of Medicine.

### Glucose and Insulin Tolerance Test

For the glucose tolerance test (GTT), mice were fasted for 12–14 h, followed by intraperitoneal injection of a 20% glucose solution (Sigma, D9434) at a dose of 1 g kg^−1^ body weight, applicable to both CD and HFD‐fed mice. Blood glucose levels were measured from tail blood using a glucometer (Roche, Switzerland) at 0, 15, 30, 60, 90, and 120 min post‐injection. For the insulin tolerance test (ITT), mice were fasted for 6–8 h and subsequently administered insulin (Lily, HI0240) at a dose of 1 U kg^−1^ for CD‐fed mice and 1.5 U kg^−1^ for HFD‐fed mice. Baseline blood glucose levels were immediately before insulin administration, with additional measurements taken at 15, 30, 60, 90, and 120 min following injection.

### Metabolic Cage Studies

Metabolic parameters were assessed using a Comprehensive Lab Animal Monitoring System (CLAMS; Columbus Instruments) equipped with open‐circuit indirect calorimetry. Mice were acclimated to the metabolic chambers for at least 24 h prior to data collection. The system continuously recorded VO_2_, VCO_2_, heat production, RER, and physical activity. In experiments involving CL316243 treatment (Sigma, C5976), HFD‐fed mice were first subjected to baseline metabolic monitoring for 24 h. Subsequently, mice received a single intraperitoneal injection of CL316243 (1 mg kg^−1^ body weight), followed by continuous metabolic measurements for an additional 24 h.

### Body Composition Analysis

Fat and lean mass were measured using an EchoMRI body composition analyzer (AccuFat‐1050) in both CD and HFD‐fed mice.

### Histological and Immunohistochemistry Analysis

Mouse adipose and liver tissues were fixed in 4% paraformaldehyde (Servicebio, G1101), followed by paraffin embedding and sectioning. H&E staining was performed according to standard protocols. Lipid droplet sizes in adipose tissue were quantified using ImageJ. Tissue sections were blocked and incubated with primary antibodies, including rabbit anti‐Adss1 antibodies (Novus, NBP1‐55524) and rabbit anti‐Ucp1 antibody (Abcam, ab10983), followed by appropriate secondary antibodies.

### AAV‐Mediated Adss1 and Gk Knockdown

To achieve adipose tissue‐specific knockdown of Adss1 and Gk, AAV‐shRNA vectors targeting these genes were constructed and synthesized by Hanbio (Shanghai, China). The target sequences are listed in the key resources table. Experiments were performed in 8‐week‐old mice. AAV was locally injected at multiple sites within each fat pad at a total viral dose of 2 × 10^10^ viral genomes (vg) per mouse, with a cumulative injection volume of 50 µL for each fat pad. Mice were sacrificed 3–4 weeks post‐injection for tissue collection to evaluate knockdown efficiency and phenotypic outcomes. For *ob/ob* mice, AAV‐Adss1 was injected into the inguinal fat pad at 6 weeks of age at a dose of 5 × 10^10^ vg per mouse, and metabolic phenotypes were assessed 4 weeks after injection. For experiments involving shGk‐AAV, local injections were administered in the inguinal fat pads of both control and Adss1^AKO^ mice. Animals were randomly divided into four groups: AAV‐Con + control, AAV‐shGk + control, AAV‐Con+ Adss1^AKO^, and AAV‐shGk + Adss1^AKO^, with each group receiving 2 × 10^10^ vg per mouse.

### Lentivirus, Adenovirus Infections, and siRNA Transfection

For gene knockdown in mouse preadipocytes and mature adipocytes, cells were transduced with either scrambled control shRNA or lentivirus expressing shAdss1 (Shanghai GeneChem Co., Ltd.) at a multiplicity of infection (MOI) of 35–50. After 12 h of viral infection, the medium was replaced with fresh culture medium, and cells were harvested after achieving full differentiation. Overexpression of Adss1 was performed following the same procedure. For Cre recombinase‐mediated gene deletion, SVF cells isolated from the iWAT of Adss1*^fl/fl^* mice were differentiated into mature beige adipocytes, then infected with either a Cre recombinase‐expressing adenovirus or a control adenovirus lacking Cre (Shanghai Obio Technology Co., Ltd.) at an MOI of 35–50. After 24 h, the culture medium was refreshed, and cells were harvested for analysis after 48 or 72 h post‐infection. For siRNA‐mediated gene silencing, mature adipocytes were transfected with siRNA (Shanghai GenePharma Co., Ltd.) using Lipofectamine RNAiMAX (Invitrogen, 13778–150) in accordance with the manufacturer's protocol. Non‐targeting siRNA was used as a negative control.

### Oil Red O Staining

Differentiated mature adipocytes were gently washed twice with pre‐chilled 1× PBS, then fixed in 4% paraformaldehyde for 15 min. After fixation, cells were rinsed twice with 1× PBS and subsequently incubated with filtered Oil Red O solution (Sigma, O1391) at RT for 15–20 min. Following staining, excess dye was removed by washing twice with deionized water, and air‐dried in a cool, dry place. Lipid accumulation was then visualized by a microscope.

### Mitochondrial Staining and Mitochondrial DNA Quantification

Mitochondria in beige adipocytes from both the control and Adss1 knockdown groups were stained following the manufacturer's protocol (Beyotime, C1048). Genomic DNA was extracted using the TIANamp Genomic DNA Kit (TIANGEN, P304). Quantitative PCR was performed to amplify mitochondrial DNA using primers specific to the mt‐ND1 gene. The primers used are listed in Table S2 (Supporting Information).

### Measurement of Hepatic Triglycerides and Serum Leptin

Approximately 50 mg of liver tissue was homogenized in lysis buffer, and hepatic TG levels were quantified using a commercial assay kit (Applygen, E1013). Total protein concentrations in the sample were determined using a BCA protein assay kit (Beyotime, P0012) to normalize the hepatic TG content. Serum leptin concentrations were measured using a Leptin Mouse/Rat ELISA kit (BioVendor R&D, RD291001200R).

### Assessment of Oxygen Consumption Rate and Fatty Acid Oxidation

The oxygen consumption rate (OCR) of mature beige adipocytes was assessed using the Seahorse XF96 metabolic analyzer, following the manufacturer's guidelines (Seahorse XF Technology, Agilent). Briefly, cells were washed and incubated for 1 h with pre‐warmed Seahorse XF DMEM (Agilent, 103575–100) supplemented with 2 mM sodium pyruvate, 25 mM glucose, and 2 mM glutamine in a 37 °C non‐CO_2_ incubator. After equilibration, the plate was loaded into the analyzer, and OCR was continuously monitored following the sequential addition of oligomycin (2 µM), carbonyl cyanide‐4‐(trifluoromethoxy) phenylhydrazone (FCCP) (1.5 µM), and rotenone/antimycin A (1 µM each) (Agilent, 103015–100). The final OCR values were normalized to total protein content per well.

For the fatty acid oxidation assay, mature beige adipocytes were pre‐incubated overnight in a substrate‐limited medium to stimulate the utilization of exogenous fatty acids. The substrate‐limited medium consisted of glucose‐, glutamine‐, and pyruvate‐free DMEM supplemented with 1 mM glutamine (Agilent, 103579–100), 0.5 mM glucose (Agilent, 103577–100), 0.5 mM L‐carnitine (Agilent, 103689–100), and 1% FBS. On the day of assay, the medium was replaced with fatty acid oxidation assay medium, consisting of Seahorse XF DMEM supplemented with 0.5 mM L‐carnitine and 2 mM glucose. Cells were washed once and incubated with this medium for 45–60 min at 37 °C in a CO_2_‐free incubator. Immediately prior to measurement, the assay medium was replaced with another 150 µL of fresh, pre‐warmed assay medium, and 30 µL of palmitate‐BSA (Agilent, 102720–100) was added to the appropriate wells. OCR was then measured following sequential injection of Etomoxir (4 µM), oligomycin (2 µM), FCCP (2 µM), rotenone (Rot, 1 µM), and antimycin A (AA, 1 µM) (Agilent, 103672–100).

### Glycerol and NEFA Measurements

The concentrations of NEFA and glycerol in the cell culture supernatants and mouse serum were quantified using commercial assay kits, following the manufacturers’ instructions (NEFA: Wako, 294–63601; Glycerol: Sigma, F6428). For in vitro experiments, mature differentiated beige adipocytes were incubated in serum‐free medium containing 2% BSA (Proliant, 50‐109‐0675) for 2 h, followed by stimulation with or without 5 mM ISO. Supernatants were collected at baseline and at 1 and 3 h post‐treatment for quantification of glycerol and NEFA levels.

For in vivo measurements, serum NEFA and glycerol levels were assessed under the following experimental conditions. (a) CL316243 stimulation: Mice were fasted for 12 h, and baseline blood samples were collected from the retro‐orbital sinus. Mice then received an intraperitoneal injection of CL316243 (1 mg kg^−1^) with additional blood samples collected at 15, 30, and 60 min post‐injection. (b) Mice were exposed to cold for 3 days with free access to water and food, and serum samples were collected thereafter. (c) Serum samples were collected from aged, HFD‐fed, and *ob/ob* mice after overnight fasting.

### Glycerol Kinase Activity Assay

Glycerol kinase activity was measured following a previously described method.^[^ ^14^ ^]^ ≈100 mg of fresh iWAT was homogenized in 300 µL of extraction buffer using a glass homogenizer. The extraction buffer contained 50 mM HEPES, 11 mM MgCl_2_ (pH 7.8), 40 mM KCl, 1 mM EDTA, and 1 mM DTT. The homogenate was then centrifuged at 15000 × g for 15–20 min at 4 °C, and the supernatant was transferred to a new microcentrifuge tube for analysis. The assay buffer was prepared with 500 µM ^3^H‐glycerol, 4 mM glycerol, 50 mM Tris‐HCl (pH 7.2), 100 mM KCl, 10 mM MgCl_2_, 2.5 mM DTT, and 5 mM ATP. For the enzymatic assay, 10 µg of protein was incubated with 50 µL of the assay buffer at 37 °C for 90 min. The reaction was terminated by adding 100 µL of stop solution (ethanol: methanol, 97:3). The reaction mixture was then filtered through a Whatman filter and air‐dried. Radioactivity retained on the filter was quantified using liquid scintillation counting.

### Quantification of Intracellular Glycerol‐3‐Phosphate

SVF cells in iWAT isolated from Adss1^fl/fl^ mice were induced to differentiate into beige adipocytes, then were infected with Cre recombinase adenovirus to achieve Adss1 knockdown. Cells were extracted using 500 µL of a cold extraction solvent composed of methanol: acetonitrile: water (containing 0.1% formic acid) in a 4:2:2 ratio. The samples were then incubated at −40 °C for 30 min, followed by 10 min at 4 °C. After centrifugation at 15 000 g for 15 min at 4 °C, the supernatant was evaporated to dryness under a gentle stream of nitrogen and reconstituted in 50 µL of 50% acetonitrile containing 1 µg mL^−1^ phenylalanine‐d5 as an internal standard, and subjected to UHPLC‐HRMS/MS analysis. Chromatographic separation was performed on a ThermoFisher Ultimate 3000 UHPLC system with a Waters BEH Amide column. Mass spectrometric detection was carried out using a ThermoFisher Q Exactive Hybrid Quadrupole‐Orbitrap Mass Spectrometer equipped with a Heated Electrospray Ionization source in negative ion mode. A calibration curve was generated by plotting the concentration of G3P standards (x‐axis) against the ratio of the peak area of G3P to that of the internal standard (y‐axis). This calibration curve was then used to calculate the G3P concentrations in the samples.

### HDAC Activity Assay

HDAC activity in mature beige adipocytes was quantified using a commercial assay kit (Abcam, ab156064). The assay was performed following the One‐Step Method, according to the manufacturer's instructions.

### RNA Isolation, Quantitative RT‐PCR, and RNA Seq

Total RNA was extracted from tissues or cells using the TRIzol reagent (Invitrogen, 15 596 018), and transcribed to cDNA using the PrimeScript RT Reagent Kit (Takara, RR047B). Quantitative RT‐PCR (qPCR) was performed with SYBR Premix Ex Taq (Vazyme, Q511‐03). Primer sequences are listed in Table S2 (Supporting Information). For RNA‐seq, high‐quality RNA samples were employed to construct mRNA libraries. Sequencing was conducted on the NovaSeq 6000 platform (Illumina, USA). Raw sequencing data were processed to quantify transcript expression and identify differentially expressed genes. RNA‐seq experiments and data analysis were performed by Sinotech Genomics Co., Ltd. (Shanghai, China).

### Western Blotting

Unless otherwise specified, proteins were extracted from cells and tissues by lysing and homogenizing in RIPA buffer supplemented with a protease inhibitor cocktail (Roche, 04693132001) and a phosphatase inhibitor cocktail (Roche, 4906845001). Protein concentrations were determined using the BCA Protein Assay Kit (Beyotime, P0012). For Western blot analysis, the following primary antibodies were used: rabbit anti‐Ucp1 (Abcam, ab10983), rabbit anti‐Adss1 (NBP1‐55524), rabbit anti‐Adss2 (Abcam, ab174848), rabbit anti‐Gk (Abcam, ab126599), rabbit anti‐HSP90 (CST, 4877), rabbit anti‐phospho‐HSL (Ser660) (CST, 45 804), rabbit anti‐HSL (CST, 4107), rabbit anti‐Acetyl‐Histone H3 (Lys27) (CST, 8173), rabbit anti‐Acetyl‐Histone H3 (Lys9) (CST, 9649), rabbit anti‐Tri‐Methyl‐Histone H3 (Lys4) (CST, 9751), rabbit anti‐Tri‐Methyl‐Histone H3 (Lys27) (CST, 9733), rabbit anti‐Histone H3 (CST, 4499), rabbit anti‐Lamin A/C (CST, 2032), rabbit anti‐Phospho‐AMPKa (Thr172) (CST, 2531), rabbit anti‐AMPKa (CST, 2532), and mouse anti‐α‐Tubulin (Sigma, T6199).

### Cell Fractionation

Cells were resuspended in 500 µL of hypotonic lysis buffer (20 mM HEPES, 1 mM EDTA, 250 mM sucrose, 1 mM PMSF, 1 mM Na_3_VO_3_, and 0.5 mM DTT, pH 7.4) and incubated on ice for 10 min. Cell lysates were centrifuged at 12000 rpm for 10 min at 4 °C. The supernatants were collected as the cytoplasmic fraction. The nuclear pellets were washed three times with the lysis buffer, then resuspended and lysed by vigorous vortexing in 50 µL of RIPA buffer supplemented with 1 mM PMSF and 1 mM Na_3_VO_3_. After 30 min of incubation on ice with occasional mixing, samples were centrifuged at 12000 rpm for 10 min at 4 °C, and the supernatants were collected as the nuclear fraction.

### Acidic Extraction of Histones

Histones were isolated using an acidic extraction method. Cells were lysed on ice for 30 min in a lysis buffer containing 10 mM KCl,10 mM HEPES,1.5 mM MgCl_2_, 0.5 mM dithiothreitol, and protease inhibitors. Hydrochloric acid was then added to the lysate to a final concentration of 0.2 M, followed by incubation at 4 °C for an additional 30 min. The mixture was centrifuged at 16000 × g for 10 min at 4 °C, and the supernatant was transferred to a fresh tube. An equal volume of 25% trichloroacetic acid was added, and the mixture was incubated at 4 °C for another 30 min. After a second centrifugation at 16000 × g for 10 min at 4 °C, the supernatant was discarded, and the histone pellet was obtained. The pellet was then washed with acetone, air‐dried, dissolved, quantified, and denatured for subsequent immunoblotting analysis.

### Targeted Metabolomic Quantification Using LC‐MS

Approximately one million mature beige adipocytes were collected for targeted metabolomic analysis. To each tube containing the cell pellet, 200 µL of prechilled 80% methanol‐water was added. The samples were sonicated at 15% power for five cycles of 5 s each. After sonication, samples were centrifuged at 18000 × g for 20 min at 4 °C, and 60 µL of the supernatant was transferred to autosampler vials for LC‐MS injection. For the detection of S‐AMP, 40 µL of the supernatant was transferred to a 96‐well plate. To each well, 20 µL of 200 mM 3‐NPH and 20 µL of 120 mM EDC were sequentially added. The mixture was incubated at 30 °C with shaking at 1450 rpm for 60 min. Following the reaction, 350 µL of ice‐cold methanol was added to dilute the samples, and then centrifuged at 4000 × g for 20 min at 4 °C. A total of 150 µL of the supernatant was transferred to a new 96‐well plate for LC‐MS analysis. Nucleotide and IMP levels were quantified using an ultra‐performance liquid chromatography‐tandem mass spectrometry (UPLC‐MS/MS) system (ACQUITY UPLC‐Xevo TQS, Waters Corp., Milford, MA, USA), while S‐AMP was detected using an Acquity‐I Xevo TQ‐S liquid chromatography‐mass spectrometry (LC‐MS/MS) system (Waters Corp., Milford, MA, USA). Chromatographic separation was performed using different columns: an AdvanceBio MS Spent Media column (2.7 µm × 2.1 mm × 100 mm) for nucleotide analysis, an Atlantis Premier BEH C18 AX column (2.1 × 100 mm, 1.7 µm) for IMP, and a BEH C18 column (1.7 µm, 2.1 × 100 mm) for S‐AMP. Column temperatures were maintained at 35 °C for IMP and nucleotides, and 40 °C for S‐AMP. Raw data files generated from UPLC‐MS/MS were processed with the MassLynx software (v4.1; Waters, Corp.) for peak integration, calibration, and quantification. All targeted metabolomic analyses were conducted by Metabo‐Profile Biotechnology (Shanghai, China).

### Chromatin Immunoprecipitation (ChIP)

ChIP assays were performed using the SimpleChIP Plus Enzymatic Chromatin IP Kit (CST, 9005s). Briefly, mature adipocytes from both the control and Cre recombinase‐mediated Adss1 knockdown groups were cultured in 10 cm dishes and crosslinked with 1% formaldehyde at RT for 10 min. The crosslinking reaction was terminated by the addition of glycine solution. Nuclei were then isolated, and chromatin was enzymatically digested with nuclease, followed by sonication to generate DNA fragments ≈200–1000 bp in Length. For immunoprecipitation, the fragmented chromatin was incubated overnight at 4 °C with either anti‐Acetyl‐Histone H3 (Lys27) or normal rabbit IgG (CST, 2729) as a negative control. The following day, Protein G magnetic beads were added to each sample and incubated with rotation at 4 °C for 2 h to capture the antibody‐bound chromatin complexes. After washing with the kit‐supplied buffers, chromatin was eluted by gentle agitation of the beads at 65 °C and 1200 rpm for 30 min. The eluted chromatin was then purified and subjected to qPCR analysis. Primer sequences used for ChIP‐qPCR are listed in Table S2 (Supporting Information).

### Immunoprecipitation

Differentiated adipocytes were washed with cold PBS and harvested using a cell scraper. The cells were resuspended in immunoprecipitation buffer (20 mM Tris, pH 7.5, 150 mM NaCl, 1% Triton X‐100, 1 mM PMSF, 1 mM Na3VO4, and 1 mM NaF), mixed thoroughly, and incubated on ice for 20 min. After centrifugation (14000 g, 10 min, 4 °C), the supernatants were collected and incubated overnight at 4 °C with Anti‐Flag magnetic beads (MedChemExpress, HY‐K0207) or with Protein A/G magnetic beads (MedChemExpress, HY‐K0202) pre‐bound to anti‐Adss1 or anti‐HDAC3 antibody. The following day, the beads were washed three times with immunoprecipitation buffer. After centrifugation (2000 g, 5 min, 4 °C), the supernatants were collected for western blot analysis.

### Echocardiographic Assessment

At 75 weeks of age, cardiac function was evaluated in both control and Adss1^AKO^ mice using a high‐resolution imaging system (Vevo2100, Visual Sonic Inc., CA). Mice were anesthetized with a mixture of isoflurane and oxygen, and then were positioned on an echocardiography pad. 2D images were acquired in the long‐axis view of the left ventricular. Key functional parameters, including fractional shortening (FS), ejection fraction (EF), ratio of peak early (E) to peak late (A) transmitral flow velocity (E/A), and the ratio of peak E‐wave velocity to peak e' velocity (E/E’), were measured using M‐mode imaging. Each measurement was averaged over three consecutive cardiac cycles to ensure accuracy.

### Statistical Analysis

Comparisons between two groups were performed using a two‐tailed unpaired Student's t‐test. For analyses involving variables, a two‐way analysis of variance (ANOVA) followed by Bonferroni's multiple comparison test was applied. All statistical analyses were conducted using GraphPad Prism 8 software. Data were presented as mean ± SEM. Statistical significance was defined as ^*^ *p* < 0.05, ^**^ *p* < 0.01, ^***^ *p* < 0.001.

## Conflict of Interest

The authors declare no conflict of interest.

## Author contributions

J.S., M.A., W.L., and S.C. contributed equally to this work. Y.Y. and X.M. designed the study. J.S., M.A., W.L., S.C., Y.S., T.H., X.L., Y.Y., N.B., F.H., X.L., R.X., J.X., and J.Z. conducted the experiments and analyzed the data. J.S. and M.A. prepared the figures and drafted the manuscript. Y.Y., X.M., and Y.L. edited and revised the manuscript. Y.Y. and X.M. approved the manuscript.

## Supporting information



Supporting Information

## Data Availability

The data that support the findings of this study are available from the corresponding author upon reasonable request.
