# Supplementary material for: Adenylosuccinate Synthase 1 Deficiency Improves Energy Metabolism by Promoting Adipose Tissue Re‐esterification via Glycerol Kinase Upregulation
Source: Adv Sci (Weinh). 2025 Oct 21;13(1):e06270. doi: 10.1002/advs.202506270 (PMC12767003; doi:10.1002/advs.202506270)
Supplement: Supplementary file 1 — Supporting Information [file ADVS-13-e06270-s001.docx]

Supporting Information

**Adenylosuccinate Synthase 1 Deficiency Improves Energy Metabolism by Promoting Adipose Tissue Re-esterification via Glycerol Kinase Upregulation**

*Jingjing Sun, Miriayi Alimujiang, Wenfei Li, Shuqing Chen, Yingying Su, Tingting Hu, Xuhong Lu, Yafen Ye, Ningning Bai, Fan Hu, Xiaoya Li, Rongrong Xu, Jun Xu,* *Jiarui Zhao, Yan Lu, Xiaojing Ma* and Ying Yang**

**Figure S1**


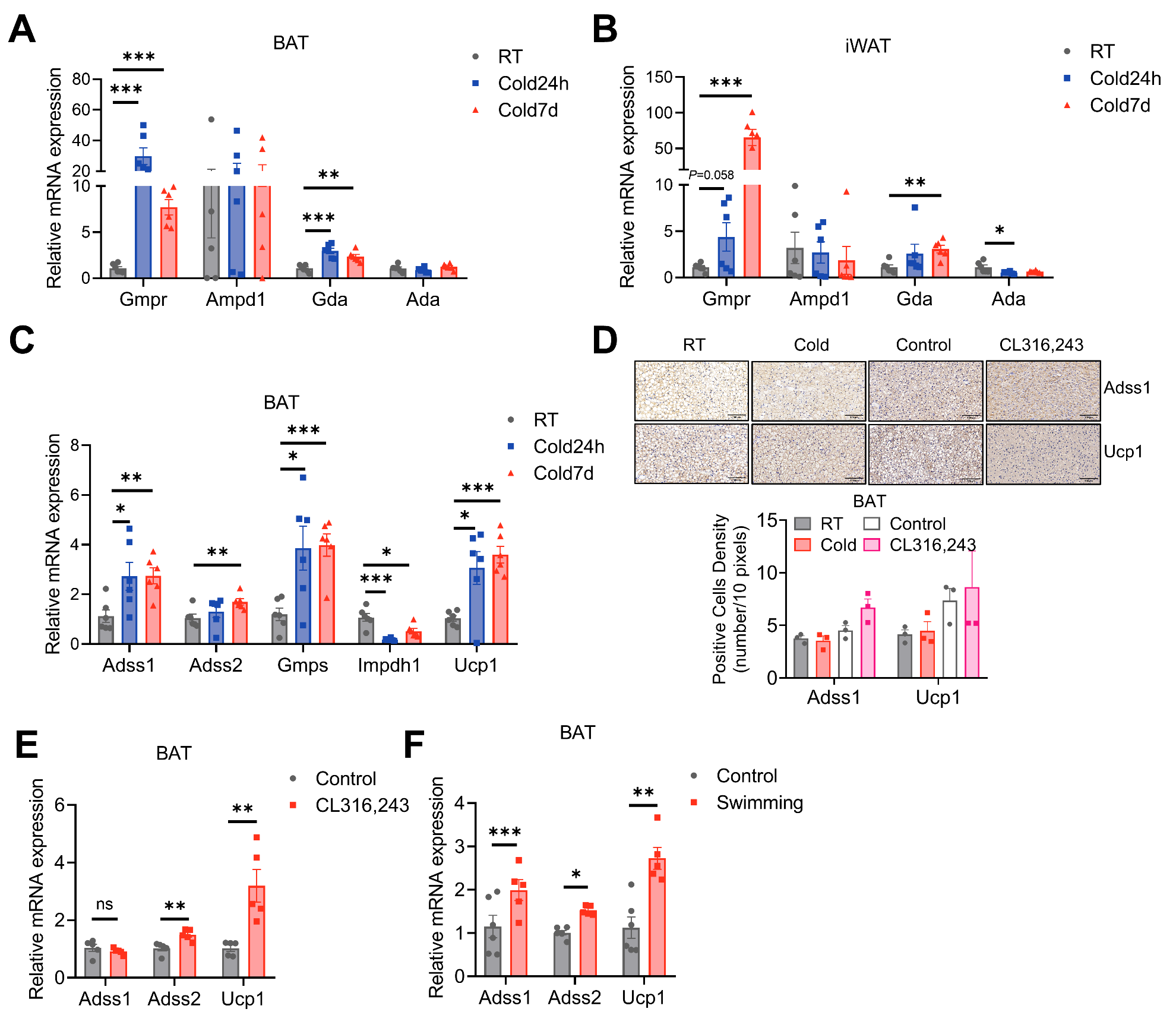


**Figure S1.** Alterated expression of purine nucleotide metabolism enzymes unpon thermogenic stimulation. A–C) mRNA expression of purine metabolism-related genes in WT mice housed at RT or exposed to cold for 24 hours or 7 days (*n* = 6). Panels show expression of purine degradation enzymes in BAT (A) and iWAT(B), and purine biosynthesis enzymes along with Ucp1 in BAT (C). D) Representative images of immunohistochemical staining of Adss1 and Ucp1 in BAT from WT mice after 7 days cold exposure or CL316,243 stimulation , scale bar=100 μm. E,F) mRNA expression of Adss1, Adss2, and Ucp1 in BAT from WT mice treated with CL316,243 for 7 days (E, *n* = 5) or swimming for 14 days (F, control *n* = 6; swimming *n* = 5). Data are presented as mean ± SEM. **P* < 0.05, ***P* < 0.01, ****P* < 0.001, Student’s *t*-test (A- F).

**Figure S2**


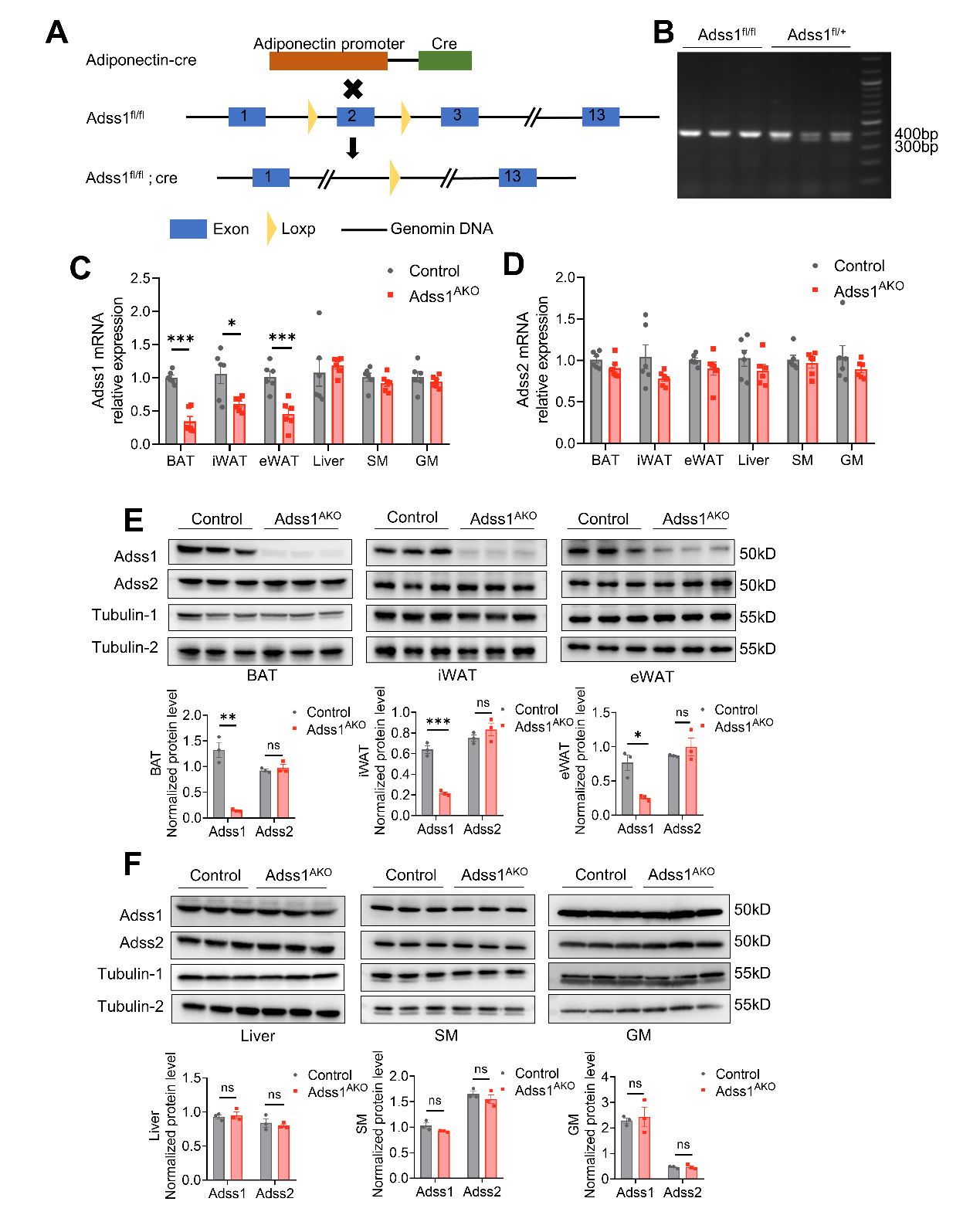


**Figure S2.** Generation and validation of Adss1^AKO^ mice. A) Schematic representation of the Adss1^fl/fl^/Adiponectin-Cre mouse model. B) Genotyping results by DNA electrophoresis showing control (Adss1^fl/fl^) and heterozygous Adss1^fl/+^ mice. C,D) Relative mRNA expression of Adss1 (C) and Adss2 (D) in the indicated tissues from control and Adss1^AKO^ mice (*n* = 6). E,F) Western blot analysis and quantification of Adss1 and Adss2 protein levels in the indicated tissues from control and Adss1^AKO^ mice (*n* = 3). Tubulin-1 and Tubulin-2 were used served as loading control of Adss1 and Adss2, respectively. Data are presented as mean ± SEM. **P* < 0.05, ***P* < 0.01, ****P* < 0.001 (Student’s t-test, C to F).

**Figure S3**

**
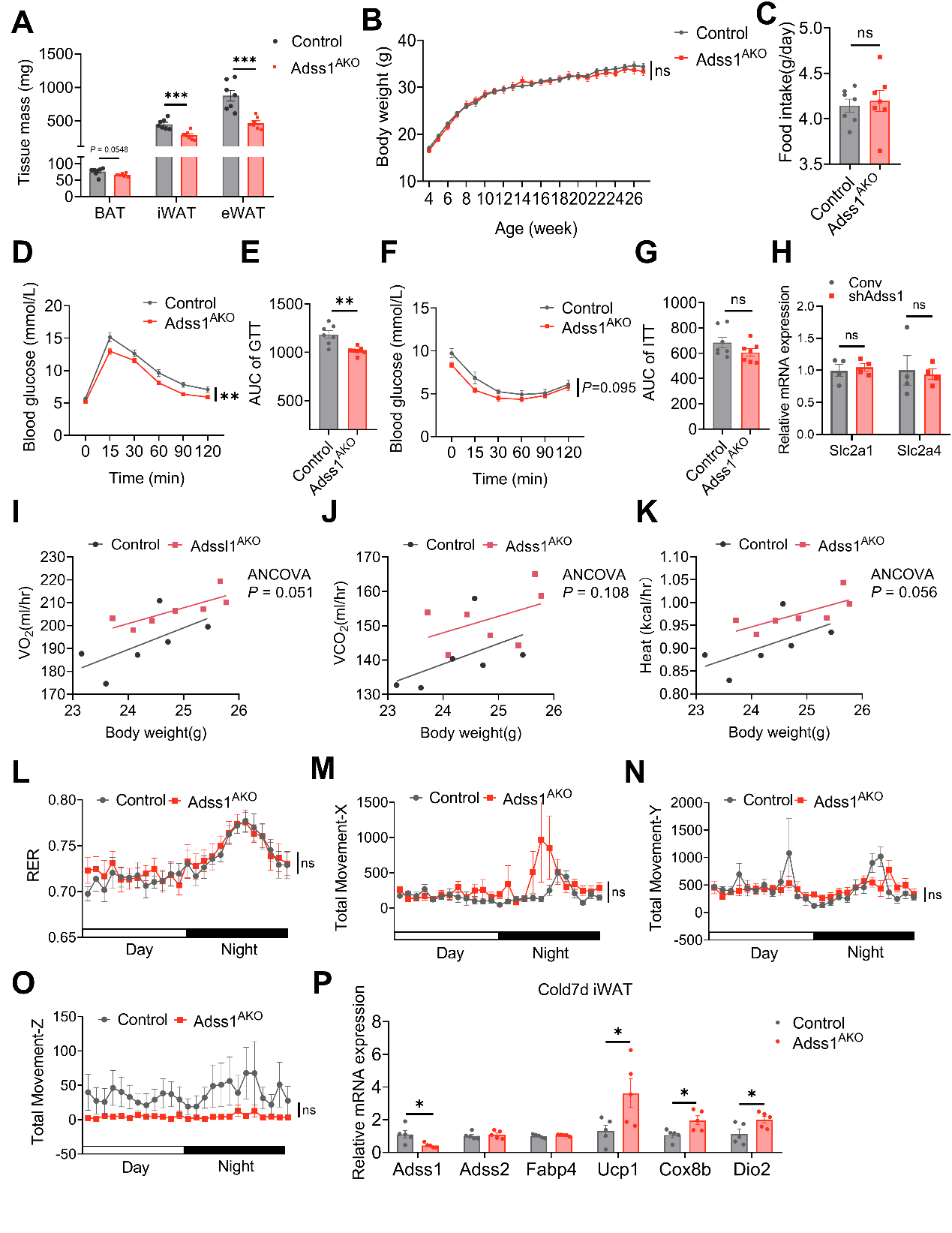
**

**Figure S3.** Metabolic phenotype of Adss1^AKO^ mice under chow diet feeding at RT and during cold exposure. A–C) Tissue weight (A), body weight (B), and food intake (C) of CD-fed control and Adss1^AKO^ mice (*n* = 7). D–G) Glucose tolerance test (GTT) (D) and corresponding AUC values (E), as well as insulin tolerance test (ITT) (F) and AUC values (G) in CD-fed control and Adss1^AKO^ mice (*n* = 7). H) Genes expression of Slc2a1 and Slc2a4 in Adss1 knockdown beige adipocytes (*n* = 4). I–O) Metabolic cage analysis of 9-week-old control (*n* = 6) and Adss1^AKO^ (*n* = 7) mice after 3 days of cold exposure. Analysis of VO_2_ (I), VCO_2_ (J), and heat production (K) after CL316,243 stimulation was conducted using ANCOVA, with body weight included as a covariate. Shown are the respiratory exchange ratio (RER) (L), total movement-X along (M), Y (N), Z (O). P) Expression levels of thermogenic marker genes in iWAT from control and Adss1^AKO^ mice after 7 days of cold exposure (*n* = 5). Data are presented as mean ± SEM. **P* < 0.05, ***P* < 0.01, ****P* < 0.001, using the Student’s *t*-test (A, C, E, G, H, and P). ANCOVA (I-K). Two-way ANOVA (B, D, F, and L-O).

**Figure S4**


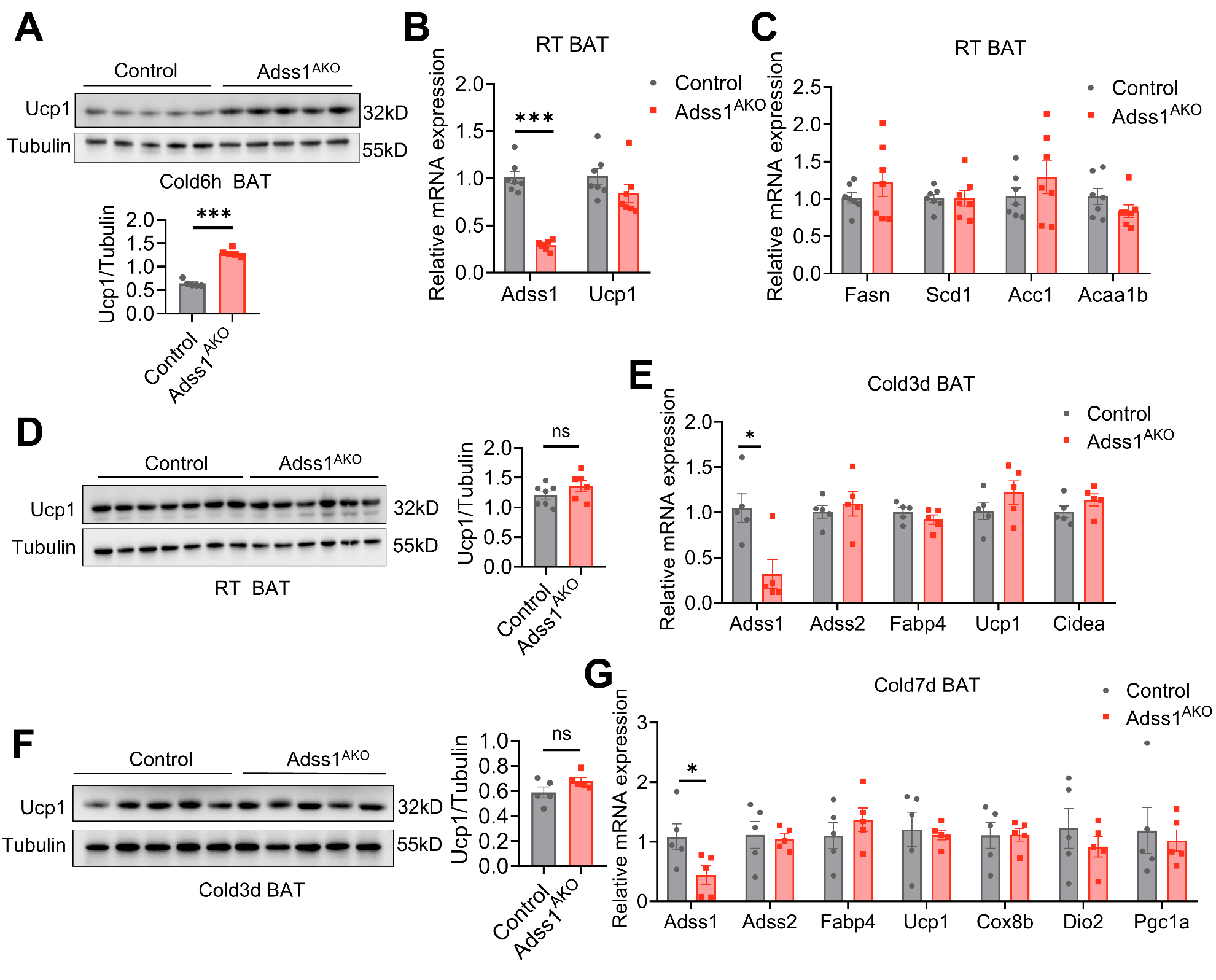


**Figure S4.** Impact of Adss1^AKO^ on the BAT function. A) Western blot analysis of Ucp1 protein levels in BAT from control and Adss1^AKO^ mice after 6 hours of cold exposure (*n* = 5). B–D) Gene expression analysis of Adss1 and Ucp1 (B) (*n* = 7), and fatty acid synthesis genes (C) in BAT (*n* = 7), and western blot analysis of Ucp1 protein at RT (D) (*n* = 7 for control, *n* = 6 for Adss1^AKO^). E,F) mRNA expression of thermogenic marker genes (E), and Ucp1 protein levels (F) in BAT from control and Adss1^AKO^ mice following 3 days of cold exposure (*n* = 5). G) Expression of thermogenic genes in BAT after 7 days of cold exposure (*n* = 5). Data are presented as mean ± SEM. **P* < 0.05, ****P* < 0.001. Statistical analysis was performed using Student’s *t*-test (A-G).

**Figure S5**


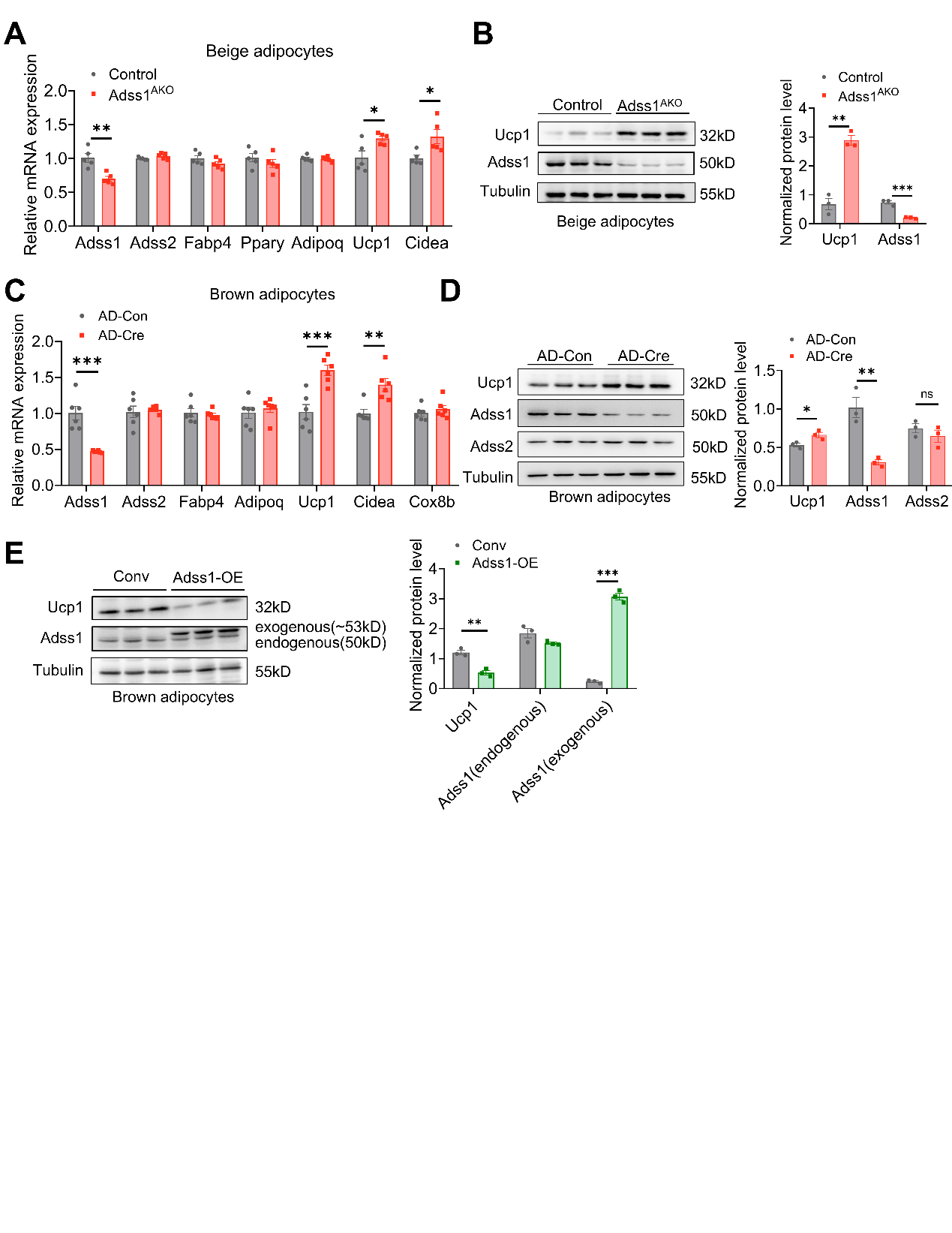


**Figure S5.** Alteration of thermogenic gene expression in beige and brown adipocytes. A-B) mRNA expression of the indicated genes (A) (*n* = 5) and protein levels of Ucp1 and Adss1 (B) (*n* = 3) in differentiated primary beige adipocytes derived from control and Adss1^AKO^ mice. C–D) Expression of the indicated genes (*n* = 6) (C) and proteins (*n* = 3) (D) in differentiated brown adipocytes from Adss1^fl/fl^ mice infected with control adenovirus (AD-Con) or Cre recombinase adenovirus (AD-Cre). E) Protein levels of Ucp1 and Adss1 in brown adipocytes with control or Adss1 overexpressing (*n* = 3). Data are presented as mean ± SEM. **P* < 0.05, ***P* < 0.01, ****P* < 0.001. Statistical analysis was performed using Student’s *t*-test (A-E).

**Figure S6**


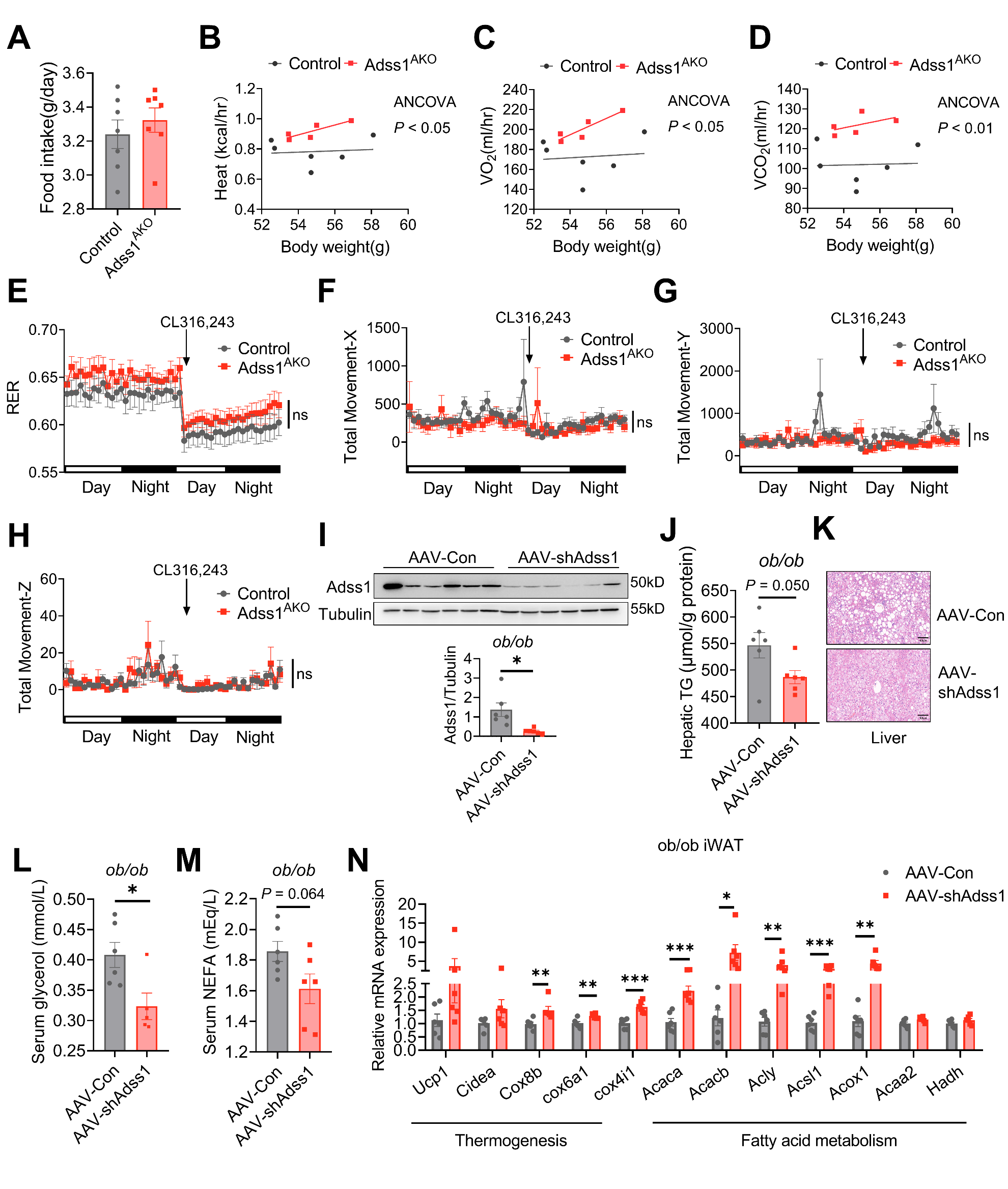


**Figure S6.** Metabolic and biochemical characterization of HFD-fed Adss1^AKO^ mice and AAV-shAdss1-treated *ob/ob* mice. A) Food intake in HFD-fed control and Adss1^AKO^ mice (*n* = 7). B–H) Metabolic cage analysis in control (*n* = 6) and Adss1^AKO^ (*n* = 5) under basal conditions and following CL316,243 stimulation. Analysis of heat production (B), VO_2_ (C) and VCO_2_ (D) after CL316,243 stimulation was conducted using ANCOVA, with body weight included as a covariate. RER (E)_._ Total movement along the X (F)_,_ Y (G), and Z (H) axes. I–N) Six-week-old male *ob/ob* mice were injected with AAV-shAdss1 or AAV-Con in iWAT. Ten weeks post-injection, mice were sacrificed for subsequent analysis, including Adss1 protein levels in iWAT (*n* = 6) (I), hepatic TG content (*n* = 6) (J), H&E staining of liver sections, scale bar = 100 μm (K), serum glycerol (*n* = 6 and 5 per group) (L), NEFA levels (*n* = 6) (M), and expression of thermogenic, fatty acid metabolism genes in iWAT of *ob/ob* mice with AAV-mediated local Adss1 knockdown in iWAT (*n* = 6) (N). Data are presented as mean ± SEM. **P* < 0.05. Student’s *t*-test (A, I, J, L-N). ANCOVA (B-D). Two-way ANOVA (E-H).

**Figure S7**


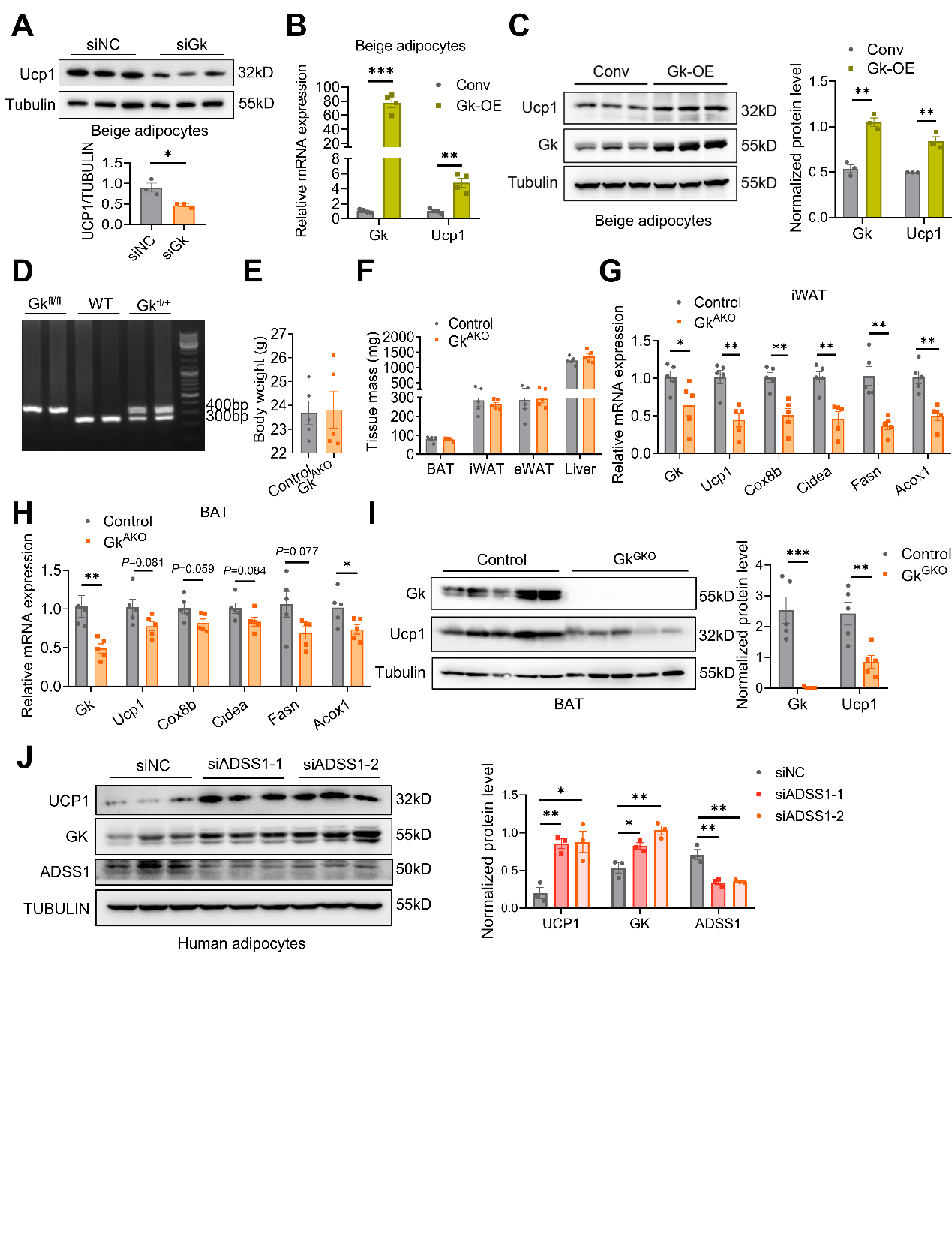


**Figure S7.** Functional analysis of Gk in adipocytes and adipose tissue-specific Gk knockout mice. A) Western blot of Ucp1 protein levels in control and Gk-knockdown beige adipocytes (*n* = 3). B) mRNA expression levels of Gk and Ucp1 following Gk overexpression in beige adipocytes (*n* = 4). C) Protein levels and quantitative analysis of Gk and Ucp1 following Gk overexpression in beige adipocytes (*n* = 3). D) Genotyping of Gk^fl/fl^, WT, and Gk^fl/+^ mice by DNA electrophoresis. E) Body weight of control and Gk^AKO^ mice (*n* = 5). F) Tissue weight of control and Gk^AKO^ mice under cold exposure for 3 days (*n* = 5). G-H) mRNA expression of thermogenic and fatty acid metabolism–related genes in iWAT (G) and BAT (H) of control and GK^AKO^ mice under cold exposure for 3 days (*n* = 5). I) Protein levels of Ucp1 and Gk in BAT of control and GK^AKO^ mice under cold exposure for 3 days (*n* = 5). J) Expression and quantitative analysis of thermogenic protein UCP1 and GK following siRNA-mediated knockdown of ADSS1 in human adipocytes (*n* = 3). Data are presented as mean ± SEM. **P* < 0.05, ***P* < 0.01, ****P* < 0.001. Student’s *t*-test (A-C, E-J).

**
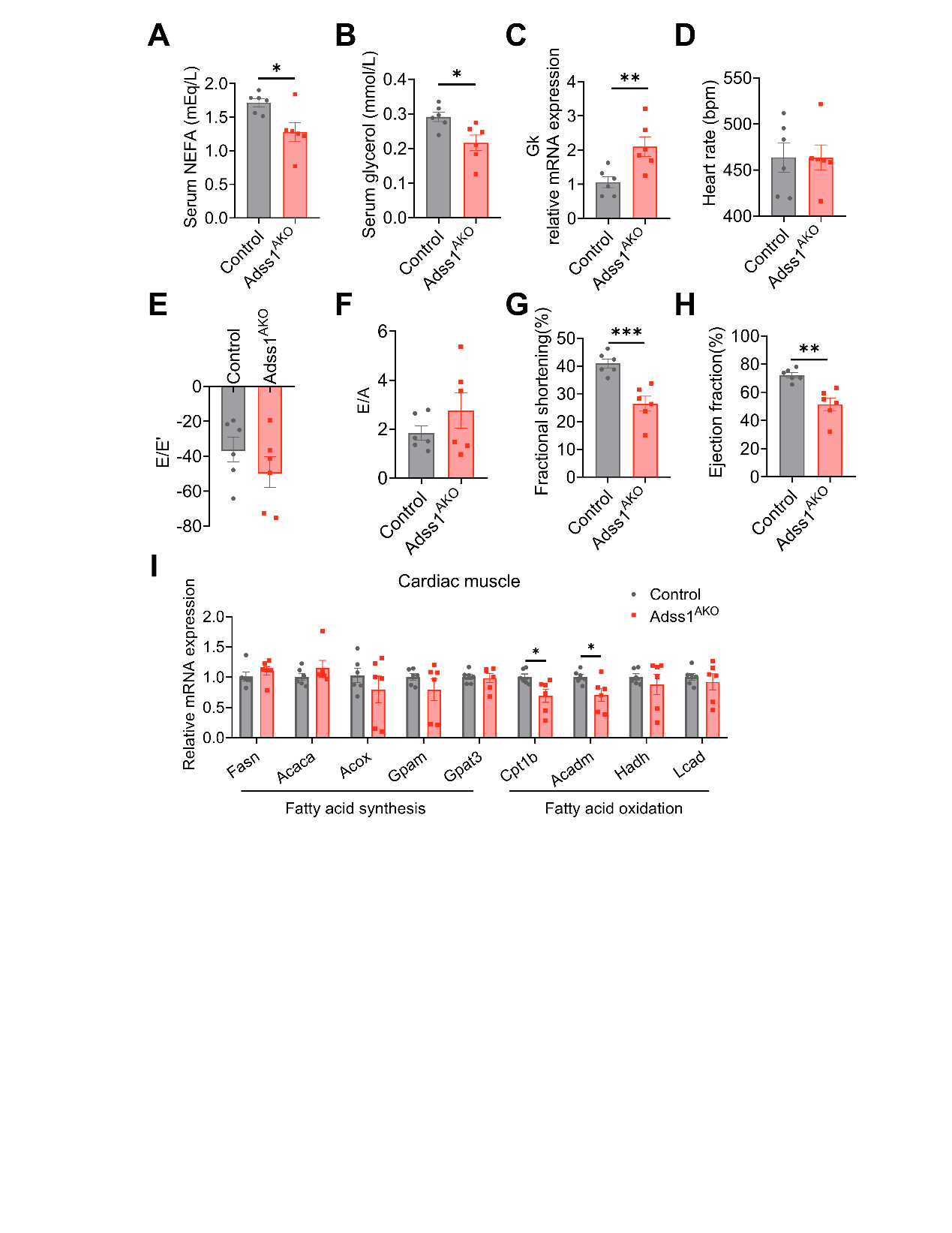
**

**Figure S8.** Metabolic and cardiac alterations in aging mice following Adss1 depletion. A–C) Metabolic assessments in aging mice, including serum NEFA (A), serum glycerol levels (B), and Gk mRNA expression in iWAT (C) (*n* = 6). D–H) Assessment of cardiac function, including heart rate (D), peak E-wave velocity/peak e' velocity (E/E’) (E), ratio between the peak early (E) and peak late (A) transmitral flow velocity (E/A) (F), fractional shortening (G), ejection fraction (H). Cardiac function analysis was assessed at 75 weeks of age, while serum and mRNA analyses were performed at 78 weeks of age in the same cohort of mice (*n* = 6). I) Expression levels of fatty acid synthesis and oxidation genes in the cardiac muscle of aging Adss1^AKO^ mice (*n* = 6). Data are presented as mean ± SEM. **P* < 0.05, ***P* < 0.01, ****P* < 0.001. Student’s *t*-test (A-I).

**Figure S9**


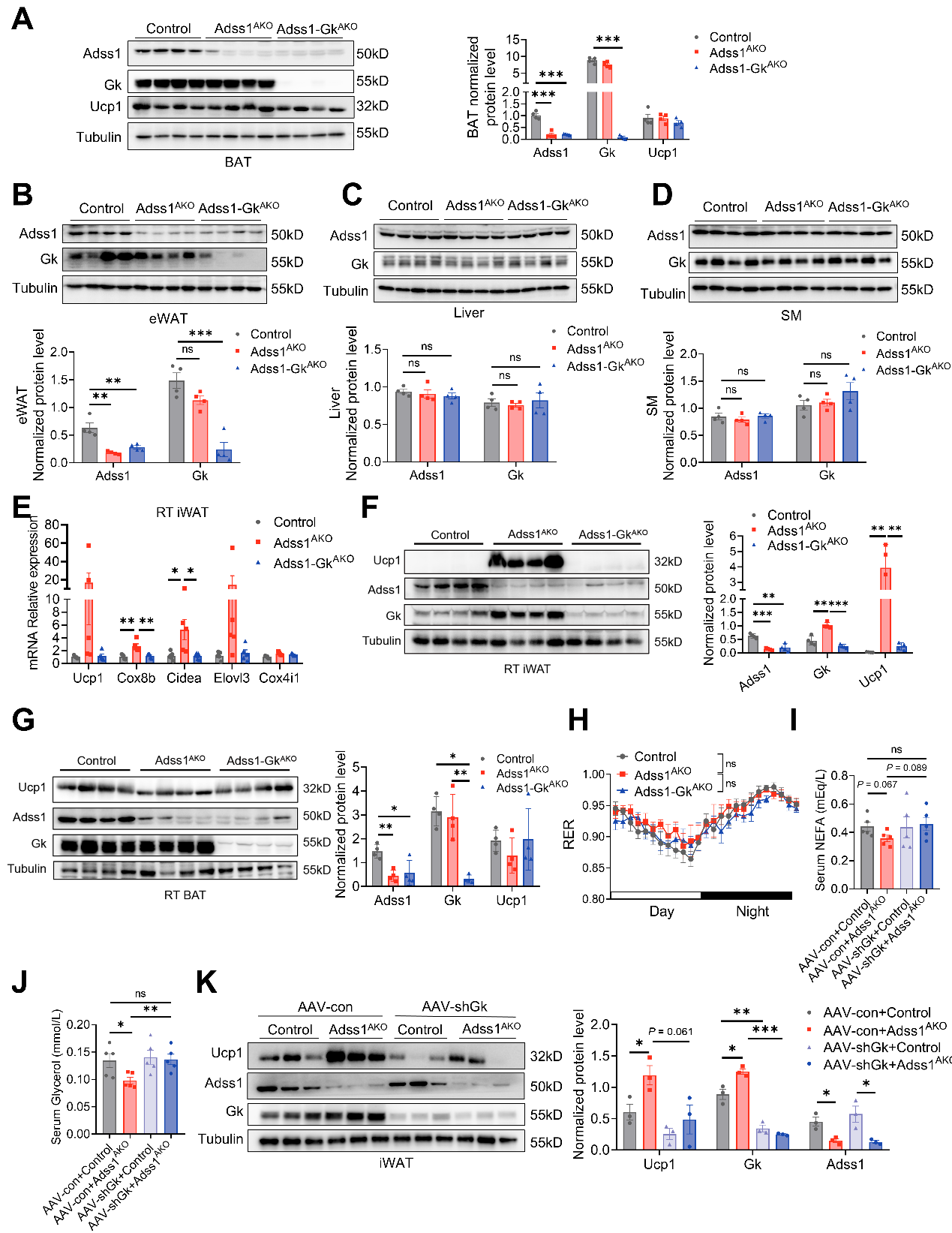


**Figure S9.** Tissue-specific validation of Adss1-Gk^AKO^ mice and the indispensable role of Gk in Adss1-mediated metabolic phenotypes. A–D) Protein expression of Adss1 and Gk in BAT (A), eWAT (B), liver (C) and SM (D) from control, Adss1^AKO^ and Adss1-Gk^AKO^ mice, confirming adipose tissue-specific knockdown (*n* = 4). E) Expression levels of thermogenesis-related genes in iWAT of Adss1^AKO^ and Adss1-Gk^AKO^ mice under RT conditions (*n* = 5). F) Western blot analysis of the indicated protein in iWAT of Adss1^AKO^ and Adss1-Gk^AKO^ under RT conditions (*n* = 4). G) Western blot analysis of the indicated protein in BAT of Adss1^AKO^ and Adss1-Gk^AKO^ under RT conditions (*n* = 4). H) RER measurements from metabolic cage in control, Adss1^AKO^, and Adss1-Gk^AKO^ mice after 3 days of cold exposure (*n* = 5). I–K) Local knockdown of Gk in iWAT: Control and Adss1^AKO^ mice received iWAT injections of AAV-Con or AAV-shGk at 6 weeks of age. After 4 weeks, the mice were exposed to cold for 3 days prior to analysis. Serum NEFA (I) and glycerol (J) levels (*n* = 5), as well as expression levels of indicated proteins in iWAT (*n* = 3) (K). Data are presented as mean ± SEM. **P* < 0.05, ***P* < 0.01, ****P* < 0.001. Student’s *t*-test (A-G, I-K). Two-way ANOVA (H).

**Figure S10**


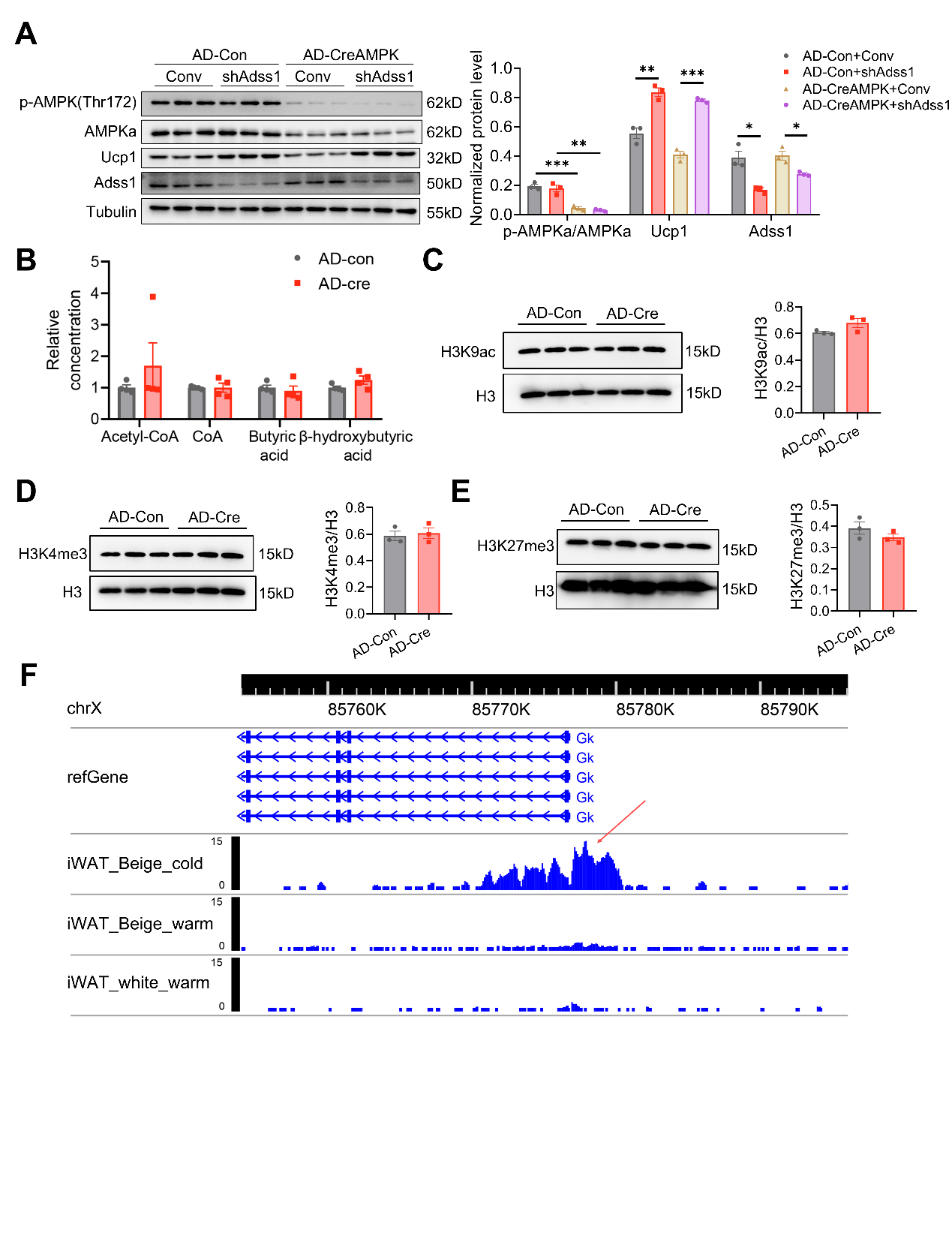


**Figure S10.** Adss1 regulates thermogenesis independently of AMPK, and cold exposure induces H3K27ac enrichment at the Gk locus. A) Western blot analysis of the indicated proteins in differentiated beige adipocytes isolated from AMPK^fl/fl^ mice, infected with control and shAdss1 lentiviruses, and subsequently transfected with Cre recombinase adenovirus to knockdown AMPK (*n* = 3). B) Targeted metabolomics analysis in beige adipocytes with Adss1 knockdown via Cre recombinase adenovirus, including acetyl-CoA, CoA , butyric acid, β-hydroxybutyric acid (*n* = 4).

C) Protein level of H3K9ac (*n* = 3) in differentiated beige adipocytes from Adss1^fl/fl^ mice infected with control adenovirus (AD-Con) or Cre recombinase adenovirus (AD-Cre). D) Western Blot analysis of H3K4me3 in Adss1-deficient beige adipocytes. E) Protein level of H3K27me3 in Adss1-deficient beige adipocytes. F) H3K27ac ChIP-seq profile upstream of Gk transcription start site (TSS) in mice exposed to different thermal conditions: 7 days of cold exposure (beige cold), 7 days of cold exposure followed by warm acclimation at 30°C for 4 weeks (beige warm) and lifelong maintenance at 30°C at all times (white warm) (Source: GSE108077). Data are presented as mean ± SEM. **P* < 0.05, ***P* < 0.01, ****P* < 0.001. Statistical analysis: Student’s *t*-test (A-E).

**Figure S11**


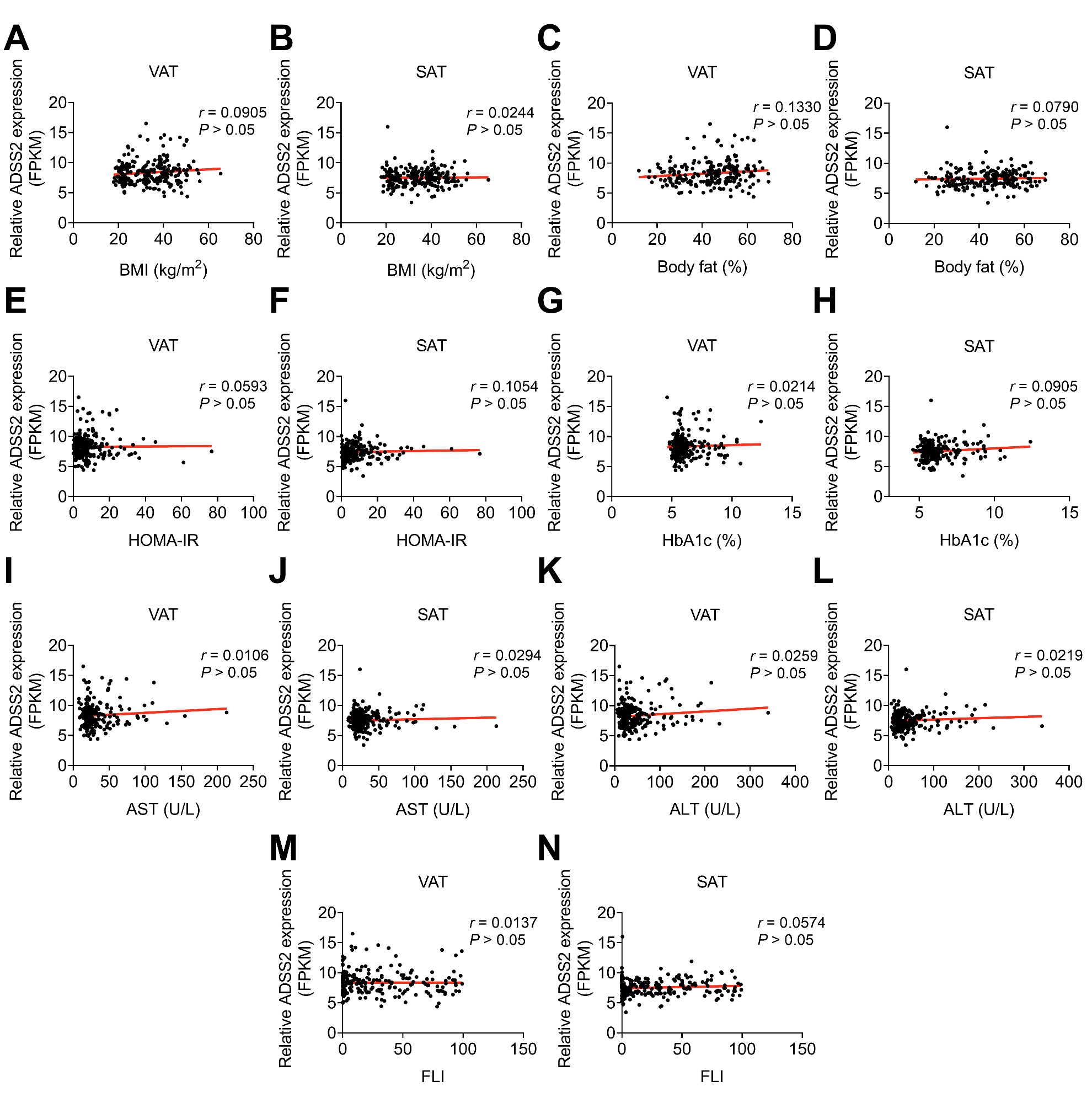


**Figure S11.** ADSS2 expression in human adipose depots shows no significant correlation with obesity and metabolic traits. Correlation analyses of ADSS2 expression in VAT and SAT with: BMI (A,B) (*n* = 236), body fat percentage (C,D) (*n* = 217), HOMA-IR (E,F) (*n* = 215), HbA1c (G,H) (*n* = 232), AST (I,J) (*n* = 236), ALT (K,L) (*n* = 236), and FLI (M,N) (*n* = 227). Spearman correlation analysis is shown by *r* values and two-tailed *P* values. Expression levels are shown as fragments per kilobase of transcript per million mapped reads (FPKM).

**Supplemental Table.**

Table S1. Primers for genotyping.

| **Target gene Forward primer sequence (5’ - 3’) Reverse primer sequence (5’ - 3’)** | | |
| --- | --- | --- |
| Adss1 LoxP | ACCACCTCATTCCTCCCTG | TCCTTGCCGTCCACCACTA |
| Gk LoxP | CTATGTTCCTGGCTGACCTGTAAC | GATGTAGGCCCAAGTTAGCAGTAAC |
|  |  |  |

Table S2. Primers for quantitative RT-PCR and ChIP-qPCR analysis.

| **Gene symbol Forward primer sequence (5’ - 3’) Reverse primer sequence (5’ - 3’)** | | |
| --- | --- | --- |
| Adss1 mouse | GGGCTCACCTTGTGTTCGAC | GGGCAGCTTTGGAGGAGTA |
| Adss2 mouse | ACACGGGGTAGAGAATTTGGA | GGTAAGGGCCAACGCAGTA |
| Ucp1 mouse | AGGCTTCCAGTACCATTAGGT | CTGAGTGAGGCAAAGCTGATTT |
| Impdh1 mouse | AAAGCCTATCTGTCCTGCGA | AGTTCTGGAGGGAGGCTGTT |
| Gmps mouse | ACACCTGGCAATGAGATTCC | GGGGGCTTTGAAGTTAGGTC |
| Gmpr mouse | GAGCTCAGACACAGCCATGA | ACTCGGCTCAACACTGAGGT |
| Ampd1 mouse | CAGAGCCTCGCTTATCCATC | ATTCTCCAGCATCTTTCCGA |
| Gda mouse | GGAATTTGATGCCCTCTTGA | GAGCTGGAGAATGGAACGAC |
| Ada mouse | AAGGAACTTCTGGAACGGCT | TCCAAGGTCTGGAAGGAATG |
| Elovl3 mouse | TCCGCGTTCTCATGTAGGTCT | GGACCTGATGCAACCCTATGA |
| Cidea mouse | TGACATTCATGGGATTGCAGAC | CATGGTTTGAAACTCGAAAAGGG |
| Dio2 mouse | CAGTGTGGTGCACGTCTCCAATC | TGAACCAAAGTTGACCACCAG |
| Cox8b mouse | GAACCATGAAGCCAACGACT | GCGAAGTTCACAGTGGTTCC |
| Cox7a1 mouse | CAGCGTCATGGTCAGTCTGT | AGAAAACCGTGTGGCAGAGA |
| Cox4i1 mouse | ATTGGCAAGAGAGCCATTTCTAC | CACGCCGATCAGCGTAAGT |
| Fabp4 mouse | AAGGTGAAGAGCATCATAACCCT | TCACGCCTTTCATAACACATTCC |
| Adipoq mouse | TGTTCCTCTTAATCCTGCCCA | CCAACCTGCACAAGTTCCCTT |
| Cebpa mouse | CAAGAACAGCAACGAGTACCG | GTCACTGGTCAACTCCAGCAC |
| Pparg mouse | TCGCTGATGCACTGCCTATG | GAGAGGTCCACAGAGCTGATT |
| Gk mouse | ACCCTCCATGCCTGAAACAA | ACCACTTTCTGGAGACTGAGTT |
| mt-ND1 | CTAACAACTATTATCTTCCTAGGAC | GATGTATAAGTTGATCGTAACGG |
| Fasn mouse | GGAGGTGGTGATAGCCGGTAT | TGGGTAATCCATAGAGCCCAG |
| Acaa2 mouse | GATCTCAAGCTGGAAGATAC | ACCTCTGCTGAGACTGCAAG |
| Hadh mouse | TCTTGACTATGTTGGACTGGATAC | AAGGACTGGGCTGAAATAAGG |
| Acadl mouse | GAAACCAGGAACTACGTGAAG | GCTGTCCACAAAAGCTCT |
| Cpt1b mouse | GCACACCAGGCAGTAGCTTT | CAGGAGTTGATTCCAGACAGGTA |
| Acadm mouse | ACCCAGATCCTAAAGTACCC | CGAAAGCAATTCCTCTGGTG |
| Acly mouse | CTGACCTTGCTGAACCCC | CCCGAGTATTCCCCGTAAT |
| Acsl1 mouse | CTGTGGGATAAACTCATCTTCC | CCTTCATAGAACTGGCAGC |
| Acc1 mouse | GATGAACCATCTCCGTTGGC | GACCCAATTATGAATCGGGAGTG |
| Acaca mouse | ATGGGCGGAATGGTCTCTTTC | TGGGGACCTTGTCTTCATCAT |
| Pck1 mouse | GGAGTACCCATTGAGGGTATCAT | GCTGAGGGCTTCATAGACAAG |
| Pck2 mouse | CAGGGTCTTATCCGCAAACT | CACATCCTTGGGGTCTGTG |
| Hdac1 mouse | AGTCTGTTACTACTACGACGGG | TGAGCAGCAAATTGTGAGTCAT |
| Hdac2 mouse | GGAGGAGGCTACACAATCCG | TCTGGAGTGTTCTGGTTTGTCA |
| Hdac3 mouse | GCCAAGACCGTGGCGTATT | GTCCAGCTCCATAGTGGAAGT |
| 36b4 mouse | AAGCGCGTCCTGGCATTGTCT | CCGCAGGGGCAGCAGTGGT |
| ChIP-Ucp1_promoter mouse | CCCACTAGCAGCTCTTTGGA | CTGTGGAGCAGCTCAAAGGT |
| ChIP-Ucp1_enhancer mouse | CTCCTCTACAGCGTCACAGAGG | AGTCTGAGGAAAGGGTTGA |
| ChIP-Gk mouse | CGGAATTCTGATCCCTACTGTGC | GACACTAGGCCAACTTCTCTGTCAA |
